# Supplementary material for: Oxytocin Shapes Spontaneous Activity Patterns in the Developing Visual Cortex by Activating Somatostatin Interneurons
Source: Curr Biol. 2021 Jan 25;31(2):322–333.e5. doi: 10.1016/j.cub.2020.10.028 (PMC7846278; doi:10.1016/j.cub.2020.10.028)
Supplement: Document S1. Figures S1–S6 and Table S1 [file mmc1.pdf]

Current Biology, Volume *31*

Supplemental Information

Oxytocin Shapes Spontaneous Activity Patterns in the Developing Visual Cortex by Activating Somatostatin Interneurons

Paloma P. Maldonado, Alvaro Nuno-Perez, Jan H. Kirchner, Elizabeth Hammock, Julijana Gjorgjieva, and Christian Lohmann

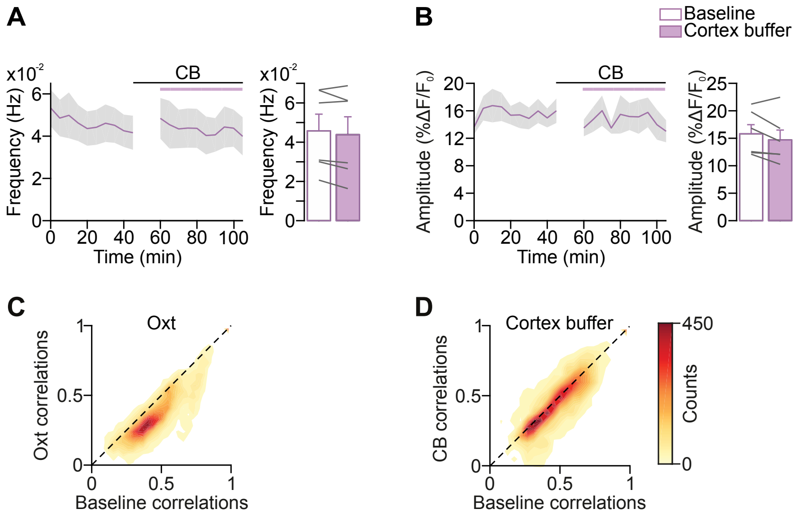

Figure S1. Effect of oxytocin and cortex buffer in two-photon imaging, related to Figure 2.

(A) Network event frequency during baseline and after cortex buffer application. Imaging resumed approximately 10 minutes after cortex buffer application. The frequency of network events was unchanged after cortex buffer application. The horizontal bar indicates significant deviations from baseline as in Figure 1 (dark shades, paired two-tailed t-test, $p < 0.05$, without multi-measurement correction). $P = 0.17$ ($n = 6$ animals, paired two-tailed t-test). CB: cortex buffer.

(B) Network event amplitude during baseline and after cortex buffer application. $P = 0.14$ (paired two-tailed t-test). Data are represented as mean ± SEM.

(C) Filled contour plot of the joint distribution of Pearson correlation coefficients. Pairwise correlations after oxytocin application plotted against baseline correlations.

(D) Pairwise correlations after cortex buffer plotted against baseline correlations.

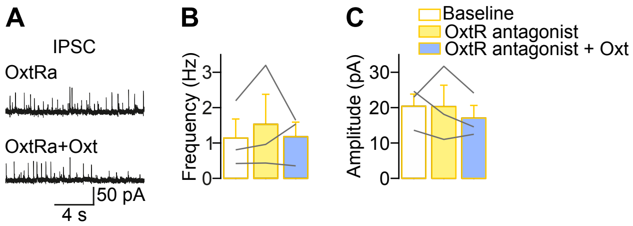

Figure S2. The oxytocin receptor mediates the increase in sIPSC frequency after oxytocin application, related to Figure 4.

(A) V1 sIPSCs in the presence of the oxytocin receptor antagonist (desGly-NH_2,d$(CH2)_5$[D-Tyr^2,Thr^4]OVT, donation from Dr. Maurice Manning, 50 μM) before (top) and after applying oxytocin (bottom).

(B) When oxytocin receptors were blocked, oxytocin failed to increase the frequency of sIPSCs. P = 0.53 (n = 3 cells, Friedman test).

(C) Amplitude of sIPSCs in the presence of the oxytocin receptor antagonist before and after oxytocin application. Data are represented as mean ± SEM.

A

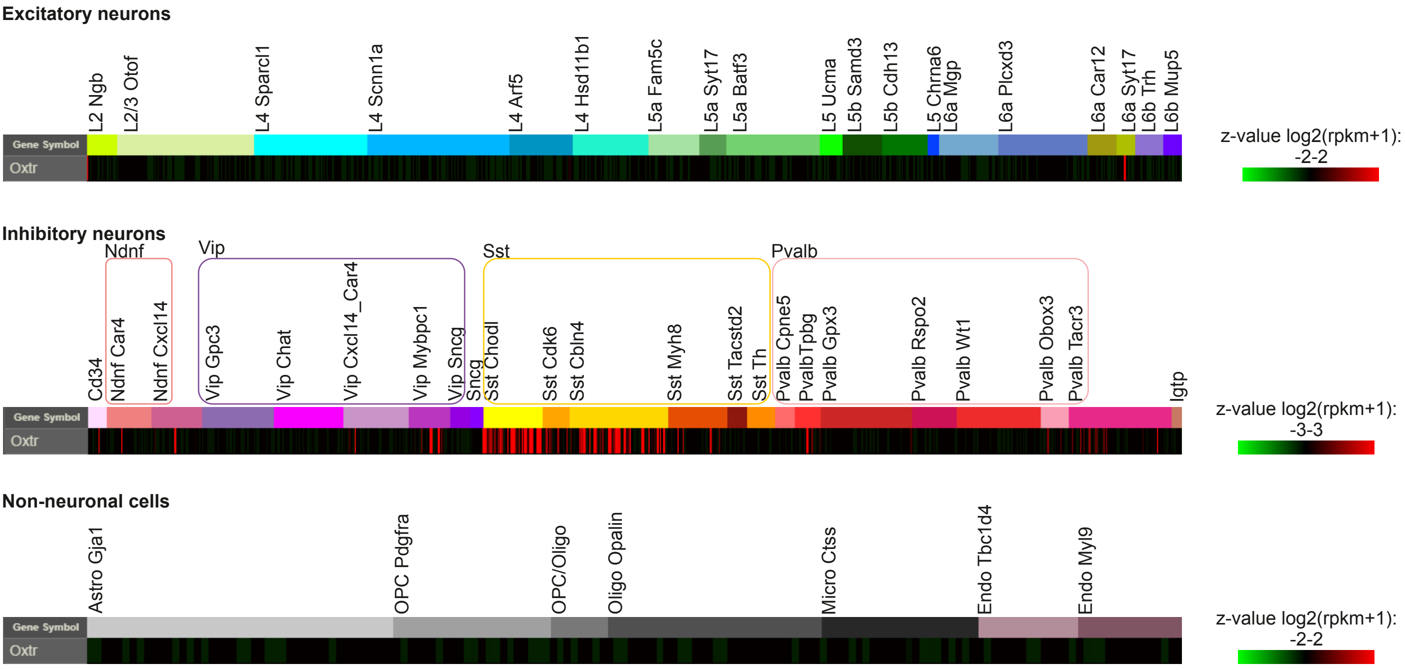

B

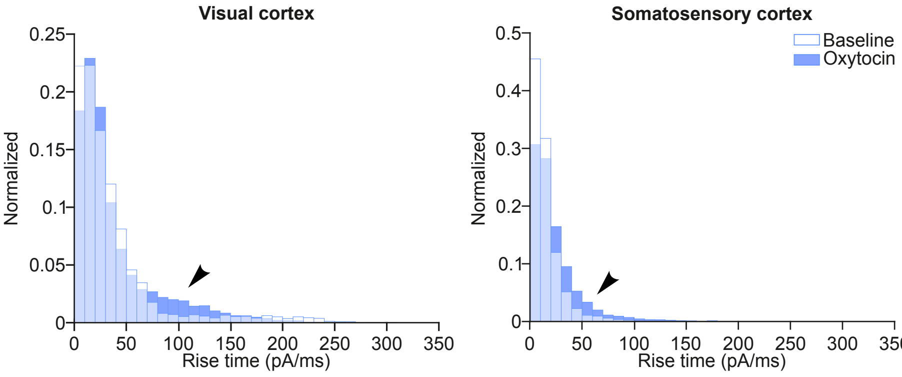

Figure S3. Evidence for oxytocin receptor activation in somatostatin^{+} interneurons , related to Figure 5.

(A) Single cell RNA-sequencing of adult visual cortex for the oxytocin receptor gene (*Oxtr*). *Oxtr* is only expressed in interneurons, in particular in those of the somatostatin-expressing type. Adapted with permission from the Allen Brain Institute [S1], Allen Brain Atlas data portal: http://casestudies.brain-map.org/celltax.

(B) Left, rise time rate histogram of V1 sIPSCs. Oxytocin shifted the histogram to the right. N = 8 cells. Kolmogorov-Smirnov test, p < 0.0001. Right, rise time rate histogram of S1 sIPSCs. As for V1, oxytocin shifted the histogram to the right as well. N = 8 cells. Kolmogorov-Smirnov test, p < 0.0001.

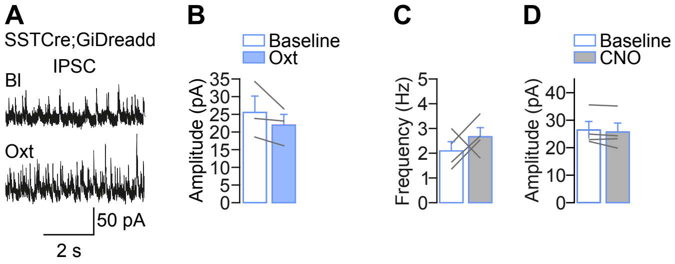

Figure S4. Oxytocin and CNO effect in SSTCre;GiDreadd and WT mice, respectively, related to Figure 5.

(A) sIPSCs before and after oxytocin application from a V1 layer 2/3 pyramidal cell of an SSTCre;GiDreadd mouse in the absence of CNO.

(B) sIPSC amplitude. N = 3 cells.

(C) sIPSC frequency before and after CNO application of WT V1 layer 2/3 pyramidal cells. P = 0.5 (n = 4 cells, Wilcoxon test).

(D) Amplitudes of sIPSCs. Data are represented as mean ± SEM.

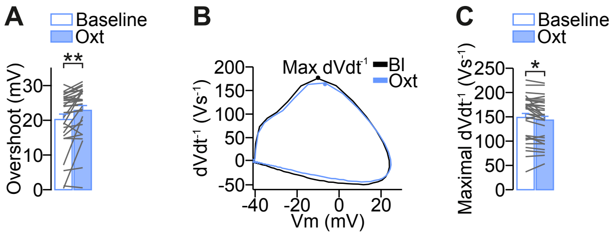

Figure S5. Oxytocin affects single action potential properties of somatostatin⁺ interneurons, related to Figure 6.

(A) AP overshoot in baseline and oxytocin conditions. **P = 0.0032 (n = 34 cells, paired two-tailed t-test).

(B) Example action potential phase plot in baseline and oxytocin conditions.

(C) Maximal time derivative of membrane voltage in baseline and oxytocin conditions. *P = 0.017 (paired two-tailed t-test). Data are represented as mean ± SEM.

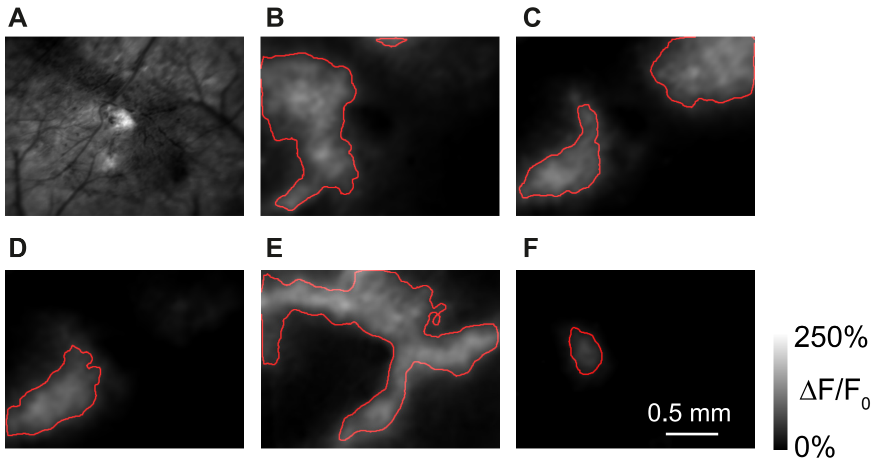

Figure S6. Automated event detection for wide-field imaging, related to Figure 1 and STAR Methods.

(A) Cortical surface showing GCaMP6s labeling.

(B-F) Spontaneous network events represented as %$\Delta F/F_0$ values in single frames and outlines generated by the automated analysis.

Physiological parameter	Value
Resting membrane potential V_m	-68.0 mV (excitatory)<br>-60.8 mV (SST^+ interneurons)<br>-56.3 mV (SST^+ interneurons after oxytocin)
Membrane time constant τ_m	84.0 ms (excitatory)<br>106.6 (SST^+ interneurons)
Spike threshold V_{th}	-36 mV (excitatory)<br>-33 mV (SST^+ interneurons)
Capacitance C_m	200 pF
Sinusoidal background input	0.05 Hz
Synaptic weight W_e, W_i	8.4 mV (AMPA)<br>-21.3 mV (GABA)

Table S1. Parameters for simulation of recurrent neural network, related to Figure 6.

Supplemental References

S1. Tasic, B., Menon, V., Nguyen, T.N., Kim, T.K., Jarsky, T., Yao, Z., Levi, B., Gray, L.T., Sorensen, S.A., Dolbeare, T., *et al.* (2016). Adult mouse cortical cell taxonomy revealed by single cell transcriptomics. Nat. Neurosci. *19*, 335–346.
