# Supplementary material for: Oxytocin Shapes Spontaneous Activity Patterns in the Developing Visual Cortex by Activating Somatostatin Interneurons
Source: Curr Biol. 2021 Jan 25;31(2):322–333.e5. doi: 10.1016/j.cub.2020.10.028 (PMC7846278; doi:10.1016/j.cub.2020.10.028)
Supplement: Document S2. Article plus Supplemental Information [file mmc2.pdf]

Article

Current Biology

Oxytocin Shapes Spontaneous Activity Patterns in the Developing Visual Cortex by Activating Somatostatin Interneurons

Graphical Abstract

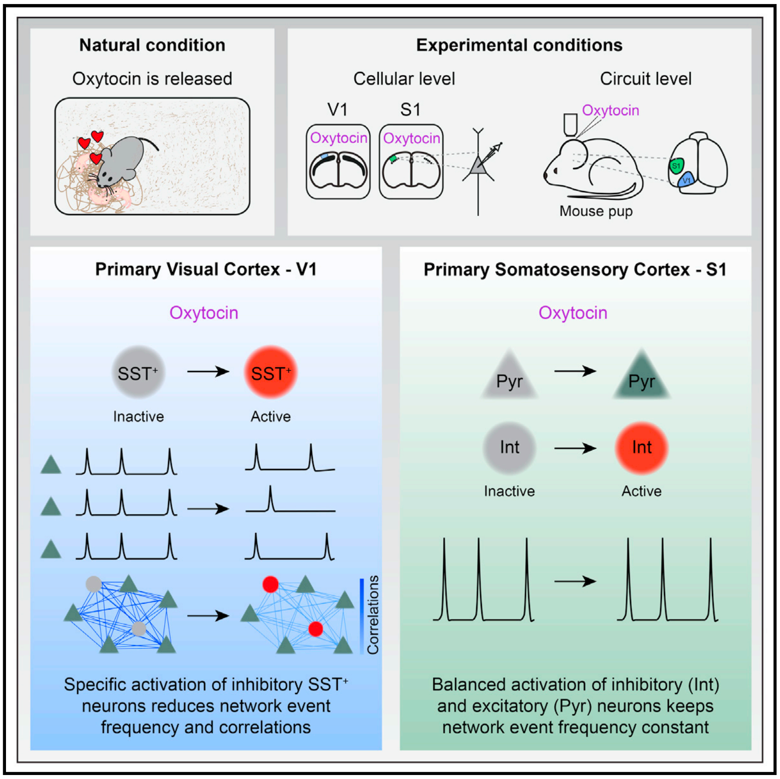

Highlights

- Oxytocin modulates spontaneous activity event patterns in the developing V1
- Oxytocin increases inhibition by activating somatostatin$^+$ interneurons
- SST$^+$ neuron activity reduces event frequency and correlations, but not amplitude
- In S1, oxytocin increases excitation and inhibition and does not modulate frequency

Authors

Paloma P. Maldonado, Alvaro Nuno-Perez, Jan H. Kirchner, Elizabeth Hammock, Julijana Gjorgjieva, Christian Lohmann

Correspondence

c.lohmann@nin.knaw.nl (C.L.)

In Brief

Maldonado et al. uncover oxytocin's role in developing sensory cortices. *In vivo*, they show that oxytocin decreases the frequency and correlations of spontaneous activity patterns in V1 by specifically activating somatostatin$^+$ interneurons. In S1, oxytocin increases both excitation and inhibition and does not affect spontaneous activity frequency.

Maldonado et al., 2021, Current Biology *31*, 322–333
January 25, 2021 © 2020 The Author(s). Published by Elsevier Inc.
https://doi.org/10.1016/j.cub.2020.10.028

CellPress
OPEN ACCESS

Current Biology

Article

Oxytocin Shapes Spontaneous Activity Patterns in the Developing Visual Cortex by Activating Somatostatin Interneurons

Paloma P. Maldonado,[1] Alvaro Nuno-Perez,[1,7] Jan H. Kirchner,[3,4] Elizabeth Hammock,[5,6] Julijana Gjorgjieva,[3,4] and Christian Lohmann[1,2,8,*]

[1]Department of Synapse and Network Development, Netherlands Institute for Neuroscience, 1105 BA Amsterdam, the Netherlands
[2]Department of Functional Genomics, Center for Neurogenomics and Cognitive Research, VU University Amsterdam, 1081 HV Amsterdam, the Netherlands
[3]Max Planck Institute for Brain Research, Computation in Neural Circuits, 60438 Frankfurt am Main, Germany
[4]TUM School of Life Sciences, Technical University of Munich, 85354 Freising, Germany
[5]Program in Neuroscience, The Florida State University, Tallahassee, FL 32306, USA
[6]Department of Psychology, The Florida State University, Tallahassee, FL 32306, USA
[7]Present address: The Department of Fundamental Neuroscience, The University of Lausanne, Rue du Bugnon 9, Lausanne 1005, Switzerland
[8]Lead Contact
*Correspondence: p.maldonado@nin.knaw.nl (P.P.M.), alvaro.nunoperez@unil.ch (A.N.-P.), jan.kirchner@brain.mpg.de (J.H.K.), hammock@psy.fsu.edu (E.H.), gjorgjieva@brain.mpg.de (J.G.), c.lohmann@nin.knaw.nl (C.L.)
https://doi.org/10.1016/j.cub.2020.10.028

SUMMARY

Spontaneous network activity shapes emerging neuronal circuits during early brain development prior to sensory perception. However, how neuromodulation influences this activity is not fully understood. Here, we report that the neuromodulator oxytocin differentially shapes spontaneous activity patterns across sensory cortices. *In vivo*, oxytocin strongly decreased the frequency and pairwise correlations of spontaneous activity events in the primary visual cortex (V1), but it did not affect the frequency of spontaneous network events in the somatosensory cortex (S1). Patch-clamp recordings in slices and RNAscope showed that oxytocin affects S1 excitatory and inhibitory neurons similarly, whereas in V1, oxytocin targets only inhibitory neurons. Somatostatin-positive (SST^+) interneurons expressed the oxytocin receptor and were activated by oxytocin in V1. Accordingly, pharmacogenetic silencing of V1 SST^+ interneurons fully blocked oxytocin's effect on inhibition *in vitro* as well its effect on spontaneous activity patterns *in vivo*. Thus, oxytocin decreases the excitatory/inhibitory (E/I) ratio by recruiting SST^+ interneurons and modulates specific features of V1 spontaneous activity patterns that are crucial for the wiring and refining of developing sensory circuits.

INTRODUCTION

In the developing brain, neuronal connections form with remarkable precision. First, axons grow to predetermined target areas guided by molecular cues. Subsequently, activity-dependent processes refine synaptic connections:[1–3] already before the senses become active, spontaneous activity drives synaptic refinement to prepare the brain for interacting with the outside world. Finally, circuits adapt to the prevalent environmental conditions through sensory-experience-driven plasticity mechanisms.

Spontaneous activity is expressed in specific patterns, and these patterns are crucial for the appropriate wiring of neurons. For example, in the developing retina, waves of spontaneous activity travel at specific speeds, in various directions, and with different wave front shapes.[4,5] Retinal waves drive highly structured activity patterns in the central visual system, including the primary visual cortex.[6–8] Perturbing these activity patterns leads to miswiring of the central visual system.[9–11]

During the period when synaptic connections are shaped by spontaneous activity, neuromodulators play an important role in the development of cortical circuits.[12–14] One of them, oxytocin, is particularly prominently expressed in sensory cortices during the first 2 postnatal weeks and decreases thereafter until the end of the 3rd postnatal week, when it reaches adult levels.[15] Similarly, oxytocin receptor ligand binding, immunolabeling, and mRNA expression in sensory cortices peak during the 2nd postnatal week and decrease thereafter.[15–17] Thus, oxytocin is most likely required for the development of sensory circuits during the 1st postnatal weeks in addition to its roles in social sensory processing in adults, where oxytocin increases the sensitivity of auditory cortex neurons to pup calls,[18] modulates odor processing in the olfactory and accessory systems,[19] and shapes social sensory perception.[20,21] Oxytocin modulates synaptic transmission in the developing forebrain. For example, oxytocin is required for cross-modal, experience-driven synaptic plasticity in the somatosensory cortex during the first 2 weeks of life.[15] Moreover, oxytocin triggers a temporary switch of

 © 2020 The Author(s). Published by Elsevier Inc.
This is an open access article under the CC BY license (http://creativecommons.org/licenses/by/4.0/).

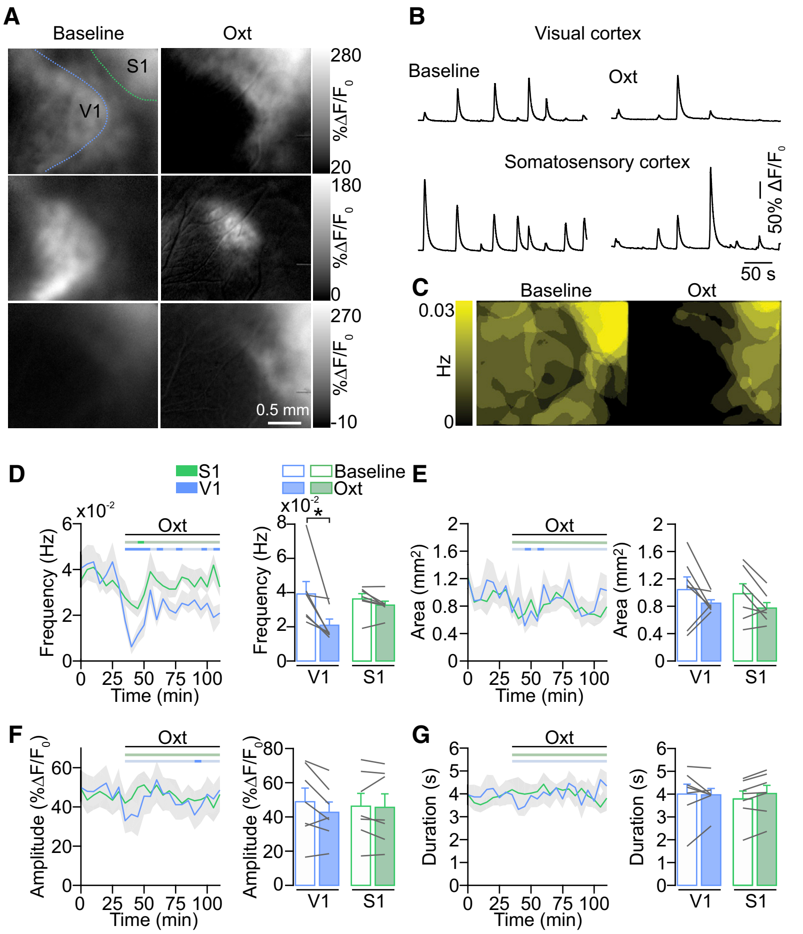

Figure 1. Oxytocin Affects Spontaneous Network Events Differentially across Sensory Cortices

(A) Wide-field calcium imaging of spontaneous activity in V1 and S1 before eye opening. (Left) Single-frame images depict network events activating V1 and/or S1 during baseline recordings before oxytocin application. (Right) Network events after oxytocin (Oxt) application are shown.

(B) Traces show fluorescent changes in V1 and S1 before and after oxytocin application.

(C) Superimposition of all network events detected during a 5-min baseline recording (left) and after oxytocin application (right). Color code indicates the frequency of the detected events. V1 activity is strongly reduced.

(D) Network event frequency in V1 and S1 during baseline and after oxytocin application. Time courses represent 5-min averages. The horizontal bars above the line plots indicate the time points when the values for each time bin differed significantly from baseline (dark shades; paired two-tailed t test; $p < 0.05$; without multi-measurement correction). *p = 0.016 (n = 7 animals; Wilcoxon test).

(E) Network event area.

(F) Network event amplitude.

(G) Network event duration.

Data are represented as mean ± SEM. See also Figure S6.

hippocampal GABA receptor action from excitatory to inhibitory.[12,22] However, it has been unclear whether spontaneous activity patterns, which drive synaptic plasticity before experience-driven refinement occurs,[23] are regulated by oxytocin as well.

Here, we asked whether oxytocin signaling shapes neuronal activity patterns in the primary visual (V1) and somatosensory cortices (S1) during the 2nd postnatal week. We found that, while in S1, oxytocin activates inhibitory and excitatory neurons similarly and does not affect the frequency of network activity, in V1, oxytocin recruits specifically somatostatin-expressing (SST$^+$) interneurons to control network activity frequency and correlation, properties known to determine the refinement of synaptic connections in V1 prior to eye opening.

RESULTS

Oxytocin Modulates Spontaneous Cortical Activity Differentially across Sensory Areas

To study the role of the neuromodulator oxytocin in developing sensory cortices, we first asked whether oxytocin receptor activation modulates large-scale spontaneous activity patterns. We used *in utero* electroporation to express the calcium sensor GCaMP6s in layer 2/3 pyramidal cells across V1, higher visual areas, and the barrel cortex. Then, we performed wide-field *in vivo* calcium imaging in lightly anesthetized pups between postnatal days (P) 9 and 13. We observed spontaneous network activity in V1, S1, and higher visual areas (Figures 1A and 1B). Frequently, network events were confined to individual sensory regions but sometimes occurred across the entire field of view. After topical application of oxytocin (1 μM) onto the cortical surface (for details, see STAR Methods), the occurrence of network events was strongly decreased in V1 but only modestly changed in S1 (Figures 1A–1D). Control application of cortex buffer without oxytocin did not change the frequency of calcium events (V1 mean frequency before cortex buffer 0.042 ± 0.002 Hz; after 0.044 ± 0.004 Hz; $p > 0.05$; n = 4; Wilcoxon test. S1 mean frequency before cortex buffer 0.042 ± 0.003 Hz; after 0.044 ± 0.004 Hz; $p > 0.05$; n = 4; Wilcoxon test). Surprisingly, the response of V1 and S1 differed significantly (percentage of change V1: −43.5% ± 7.9%; S1: −7.9% ± 5.2%; p = 0.0026; n = 7; unpaired two-tailed t test). Network event area, amplitude, or duration was not affected (Figures 1E–1G).

Oxytocin Desynchronizes Spontaneous V1 Network Activity

To evaluate how oxytocin modulates the activity of individual neurons in the developing cortical network, we performed *in vivo* two-photon calcium imaging in V1 of lightly anesthetized neonatal mice. Layer 2/3 cells were labeled with the calcium indicator Oregon Green BAPTA-1 (OGB-1) by bolus loading.[24] Oxytocin application decreased the frequency of calcium events transiently (Figures 2A–2C) without affecting their amplitude (Figure 2D), in line with our wide-field experiments. In control experiments, where we applied cortex buffer without oxytocin, frequency and amplitude were unaffected (Figures S1A and S1B).

Because the correlational structure of spontaneous network activity determines its role in network refinement,[25] we also investigated whether oxytocin affected the pairwise correlations between spontaneously active neurons. We observed that oxytocin application decreased the mean Pearson correlation coefficients across pairs of neurons (Figures 2E and 2F), whereas cortex buffer applications did not affect such correlations (Figures 2E and 2F). Next, we explored changes in correlations at the level of individual neurons after oxytocin application in more detail. We plotted pairwise correlations for all experiments during baseline against the correlations after oxytocin or cortex buffer application (Figures S1C and S1D). Again, we observed that oxytocin, but not cortex buffer application, strongly decreased correlations across the entire population. There were essentially no neuronal pairs that showed increased correlations (Figure S1C). We further found that oxytocin had a subtractive effect on neuronal correlations (Figures 2G and 2H), such that more distal pairs of neurons were proportionally decorrelated more strongly by oxytocin than nearby neuronal pairs (Figures 2I and 2J). Finally, we investigated whether the changes in correlations between all neurons occurred uniformly or whether specific pairs of neurons underwent changes in their correlations. To address this question, we first computed the correlation matrices at different time points (Figure 2K). Interestingly, we saw that, during late oxytocin, when the frequency of calcium events had returned to baseline (mean baseline: 0.032 ± 0.004 Hz; mean 125–135 min: 0.027 ± 0.003 Hz; $p = 0.14$; paired two-tailed t test), the correlation matrices exhibited sustained structural differences in the correlational structure of subsets of cells (Figure 2K, inset). To quantify these differences, we computed the mean squared distance (MSD) between the baseline matrix and the matrices computed at varying time points and found that networks treated with oxytocin exhibited a sustained higher distance compared to baseline ($p = 0.038$, repeated-measures one-way ANOVA; mean baseline: 0.1 ± 0.04; mean 60–70 min: 6.36 ± 1.56, $p = 0.007$; mean 90–100 min: 4.52 ± 1.12, $p = 0.008$; mean 125–135 min: 2.94 ± 0.6, $p = 0.009$; post hoc paired two-tailed t test). Conversely, the MSD was unchanged with cortex buffer application ($p = 0.19$; repeated-measures one-way ANOVA; Figure 2L). These oxytocin-induced changes in network activity patterns may reflect differences in functional connectivity between the imaged neurons or their shared inputs, because response correlations are indicative of strong synaptic connections in the adult and probably common feedforward inputs in the developing visual cortex.[26,27]

Oxytocin Affects the E/I Ratio Differentially across Sensory Areas

To investigate whether the differential modulation of V1 and S1 by oxytocin as described above (Figure 1) can be explained by differences in the distribution of the oxytocin receptor in these areas, we used RNAscope to detect *Oxtr*, *Scl17a7* (coding for VGLUT1), and *Gad1* mRNA in the cortex at P10. We found that *Oxtr* was expressed in V1 as well as in S1 (Figure 3). However, in V1, *Oxtr* co-localized almost exclusively with the *Gad1* signal (Figures 3B–3D), whereas in S1, the *Oxtr* signal co-localized with both the VGLUT1 and *Gad1* signal (Figures 3F–3H). These observations suggested that differences between V1 and S1 in oxytocin receptor expression on the cellular level could explain oxytocin's differential effect in these areas. To test this idea functionally, we examined how oxytocin regulated excitatory and inhibitory synaptic transmission in V1 and S1. Whole-cell patch-clamp recordings of layer 2/3 pyramidal neurons in slices from V1 showed that oxytocin affected neither the frequency nor the amplitude of spontaneous excitatory postsynaptic currents (sEPSCs) (Figures 4A–4C). Next, we measured spontaneous inhibitory postsynaptic currents (sIPSCs) at the reversal potential of glutamate-receptor-mediated currents. We found that the frequency of sIPSCs, in contrast to that of sEPSCs, was strongly increased after oxytocin bath application (Figures 4D and 4E). The amplitude of sIPSCs was unaffected (Figure 4F). To test whether this increase in frequency was mediated by the specific activation of the oxytocin and not the vasopressin 1A receptor,[28] which can be activated by oxytocin as well,[29] we blocked the oxytocin receptor using its specific antagonist OTA.[30] OTA prevented the oxytocin-mediated increase in sIPSC frequency entirely (fold-change oxytocin only, Figure 4E: 4.95 ± 1.53; oxytocin + OTA, Figure S2: 0.98 ± 0.32; $p = 0.012$; unpaired two-tailed Mann-Whitney test), demonstrating that this effect was mediated by the oxytocin receptor. Thus, activation of the oxytocin receptor increased the inhibitory tone in V1 dramatically but did not affect excitatory synaptic transmission.

In contrast to V1, in S1, oxytocin bath application increased sEPSC frequency in layer 2/3 pyramidal cells (Figures 4H and 4I). Again, sEPSC amplitude was unaffected (Figure 4J). The frequency of sIPSCs was increased but less pronounced than in V1 (Figures 4K and 4L); amplitudes were unaffected (Figure 4M). Thus, oxytocin shifted the E/I ratio in V1 toward inhibition (Figure 4G; $p < 0.001$; repeated-measurement two-way ANOVA) but did not affect E/I significantly in S1 (Figure 4N). These experiments suggested that differences in the magnitude of inhibitory versus excitatory synaptic activity modulation accounted for the differences in the effect of oxytocin on spontaneous network activity in V1 versus S1.

Oxytocin Targets SST$^+$ Interneurons in the Developing V1

Because we observed that oxytocin shaped spontaneous activity patterns effectively in V1, but not in S1, we focused next on the cellular mechanism of oxytocin-mediated facilitation of inhibition in V1. First, we measured miniature IPSCs (mIPSCs) before and after oxytocin bath application and found that mIPSC frequency and amplitude were unaffected by oxytocin (Figures 5A–5C). This indicated that the number of inhibitory synapses, the density of postsynaptic receptors, or changes in the presynaptic release machinery could not explain the increase in sIPSC frequency described above.

To search for alternative causes of the increased inhibitory activity, we performed voltage-clamp recordings in inhibitory interneurons. We used two mouse lines expressing TdTomato to target inhibitory neurons in general (GAD2-Cre;Rosa26-TdTomato) or somatostatin-expressing interneurons specifically (SST-Cre;Rosa26-TdTomato). We focused on SST$^+$ interneurons for four reasons: (1) RNA sequencing (RNA-seq) data in adulthood showed that V1 oxytocin receptors are expressed primarily in SST$^+$ interneurons (Figure S3A; Allen Brain Atlas data portal: http://casestudies.brain-map.org/celltax).[31] (2) We found

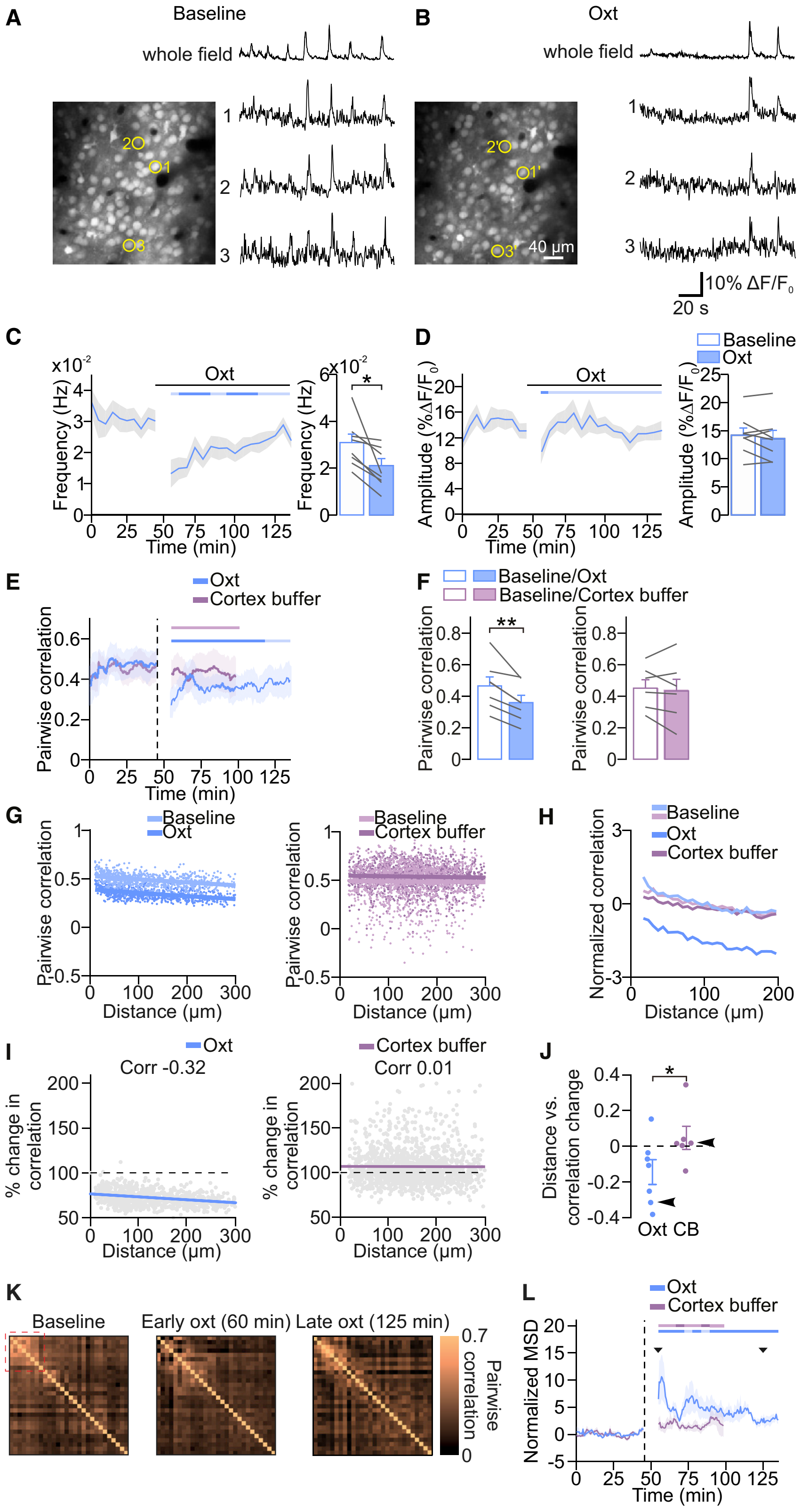

Figure 2. Oxytocin Desynchronizes Network Activity in V1

(A and B) V1 network activity before and after oxytocin application. (Left) Layer 2/3 neurons labeled with the calcium indicator Oregon Green-BAPTA 1 are shown. Traces show fluorescent changes of three example neurons and the average activity across all cells.

(C) Network event frequency during baseline and after oxytocin application. Imaging resumed approximately 10 min after oxytocin application. The frequency of network events was reduced after oxytocin application. The horizontal bar indicates significant deviations from baseline as in Figure 1 (dark shades; paired two-tailed t test; $p < 0.05$; without multi-measurement correction). *$p = 0.015$ ($n = 8$ animals; paired two-tailed t test).

(D) Network event amplitude during baseline and after oxytocin application.

(E) Time course of pairwise Pearson correlation coefficients before and after oxytocin and cortex buffer application. Dashed vertical line indicates the time point of oxytocin or cortex buffer application. Time course represents averages of sliding 7-min window (for details see STAR Methods). The horizontal bar indicates significant deviations from baseline as in Figure 1 (dark shades; paired two-tailed t test; $p < 0.05$; without multi-measurement correction).

(F) Mean correlations after oxytocin or cortex buffer control applications. **$p = 0.003$ ($n = 7$ for oxytocin; $n = 6$ for cortex buffer; paired two-tailed t test).

(G) Pairwise correlations plotted against interneuronal distance for an example of oxytocin (left) and cortex buffer (right) conditions.

(H) Mean pairwise correlations in oxytocin and cortex buffer condition. Note that baseline condition and cortex buffer display the same profile.

(I) Change in pairwise correlations plotted against interneuronal distance for an example of oxytocin (left) and cortex buffer (right) conditions. Dashed lines indicate zero change in correlations. Colored lines indicate linear fits (left, $p < 10^{-10}$; right, $p = 0.47$; paired two-tailed t test).

(J) Pearson correlation coefficients between pairwise distances and percentages of change in correlations for all animals in oxytocin and cortex buffer condition. Arrowheads indicate the examples shown in (I). *$p = 0.037$ ($n = 7$ for oxytocin; $n = 6$ for cortex buffer; unpaired two-tailed t test).

(K) Correlation matrices computed baseline, early oxytocin (60 min), and late oxytocin (125 min). Red dotted square indicates an example of a subgroup of neurons that undergo structural changes.

(L) Normalized mean squared distances (MSDs) between the baseline matrix and the correlation matrices computed from a sliding 7-min window (see STAR Methods for details) as function of time for oxytocin and cortex buffer conditions.

Data are represented as mean ± SEM. See also Figure S1.

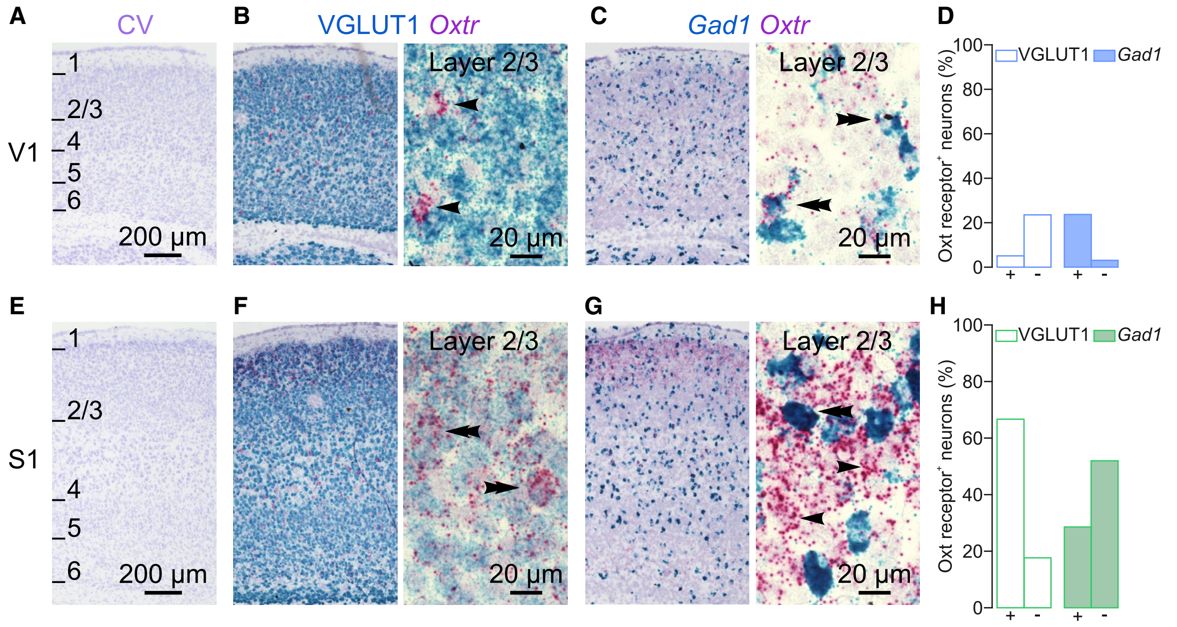

Figure 3. Oxytocin Receptor mRNA Is Differentially Expressed across Sensory Cortices

(A) Cresyl violet Nissl staining of a sagittal section from V1 at low magnification. Numbers indicate cortical layers.

(B) Dual-color RNAscope staining of VGLUT1 and *Oxtr* mRNA in a V1 sagittal section. Left: low magnification is shown. Right: high magnification is shown. Note that the *Oxtr* signal was largely non-overlapping with the VGLUT1 signal (arrowheads indicate VGLUT1^{-}/*Oxtr*$^{+}$ neurons).

(C) Dual-color RNAscope staining of *Gad1* and *Oxtr* mRNA in a V1 sagittal section. Left: low magnification is shown. Right: high magnification is shown; note that the *Oxtr* signal co-localized with the *Gad1* signal (double arrowhead *Gad1*$^{+}$/*Oxtr*$^{+}$) and not with *Gad1*$^{-}$ neurons.

(D) Quantification.

(E) Cresyl violet Nissl staining of a sagittal section from S1 (same section as in A) at low magnification. Numbers indicate cortical layers.

(F) Dual-color RNAscope staining of VGLUT1 and *Oxtr* mRNA in an S1 sagittal section. Left: low magnification is shown. Right: high magnification is shown; note the superposition of the two signals (double arrowheads, VGLUT1^{+}/*Oxtr*$^{+}$).

(G) Dual-color RNAscope staining of *Gad1* and *Oxtr* mRNA in an S1 sagittal section. Left: low magnification is shown. Right: high magnification is shown; note that the *Oxtr* signal co-localized with both *Gad1*$^{+}$ and *Gad1*$^{-}$ signal (double arrowheads indicate *Gad1*$^{+}$/*Oxtr*$^{+}$; arrowhead indicates *Gad1*$^{-}$/*Oxtr*$^{+}$ neuron).

(H) Quantification.

here that, already at P10, oxytocin receptor mRNA is expressed almost exclusively in SST^{+} interneurons in layer 2/3 of V1 (Figure 5D). (3) Decreases in network correlations similar to those described above can be induced by SST^{+} interneuron-mediated lateral inhibition.[32,33] (4) We observed here that the rise rate of V1 and S1 sIPSCs increased in response to oxytocin application (Figure S3B). Because the sIPSC rise rate is larger for synapses that are located distally in the dendritic tree, this result indicated that oxytocin preferentially increased the frequency of inhibitory inputs at distal dendrites, which receive the majority of SST^{+} interneuron inputs.[34]

Therefore, we investigated the effect of oxytocin on SST^{+} interneurons. We recorded from SST^{+} neurons in voltage-clamp mode at −60 mV and blocked NMDA, AMPA, and GABA$_A$ receptor-mediated currents using D-AP5, NBQX, and SR95531, respectively, to prevent oxytocin-mediated network effects. In this configuration, we recorded oxytocin-mediated inward currents in almost all SST^{+} neurons (83%; amplitude: −27 ± 16 pA; Figures 5E–5G). Oxytocin did not trigger inward currents in any of the SST^{-} neurons in slices from the same SST-Cre;Rosa26-TdTomato mice. We concluded that oxytocin triggered depolarizing inward currents specifically in SST^{+} interneurons. Accordingly, we observed oxytocin induced inward currents in 29% of GAD2^{+} interneurons (Figure 5G), which is similar to the proportion of SST^{+} neurons within the entire population of V1 interneurons (23%).[35]

Together, these results indicated that oxytocin mediated the increase in inhibitory synaptic activity in V1 through activation of SST^{+} interneurons. To test this idea directly, we asked whether specifically downregulating SST^{+} interneurons might prevent the oxytocin-induced increase in overall inhibition (Figure 5H). We performed voltage-clamp recordings of layer 2/3 neurons in acute slices from V1 of transgenic neonatal mice where SST^{+} interneurons expressed inhibitory designer receptors activated by designer drugs (iDREADDs) (SSTCre;Gi-Dreadd). Bath application of CNO, the activator of iDREADDs, did not affect the baseline frequency of sIPSCs (Figures 5I and 5J), suggesting that SST^{+} interneurons were only sparsely active in our slice preparation. Then, we applied oxytocin and found that it did not increase sIPSC frequency in the presence of CNO (Figures 5I and 5J), in contrast to our previous results in slices from wild-type (WT) mice (Figure 4E; fold-change WT: 4.95 ± 1.53; iDREADD: 0.95 ± 0.07; $p < 0.0001$; Mann-Whitney test). Additional control experiments showed that oxytocin did increase the frequency of sIPSCs in neurons from iDREADD-expressing animals in the absence of CNO, but not their amplitude (Figures 5J, inset; S4A; and S4B), and that CNO by itself did not change the sIPSC frequency (Figures S4C and S4D). These

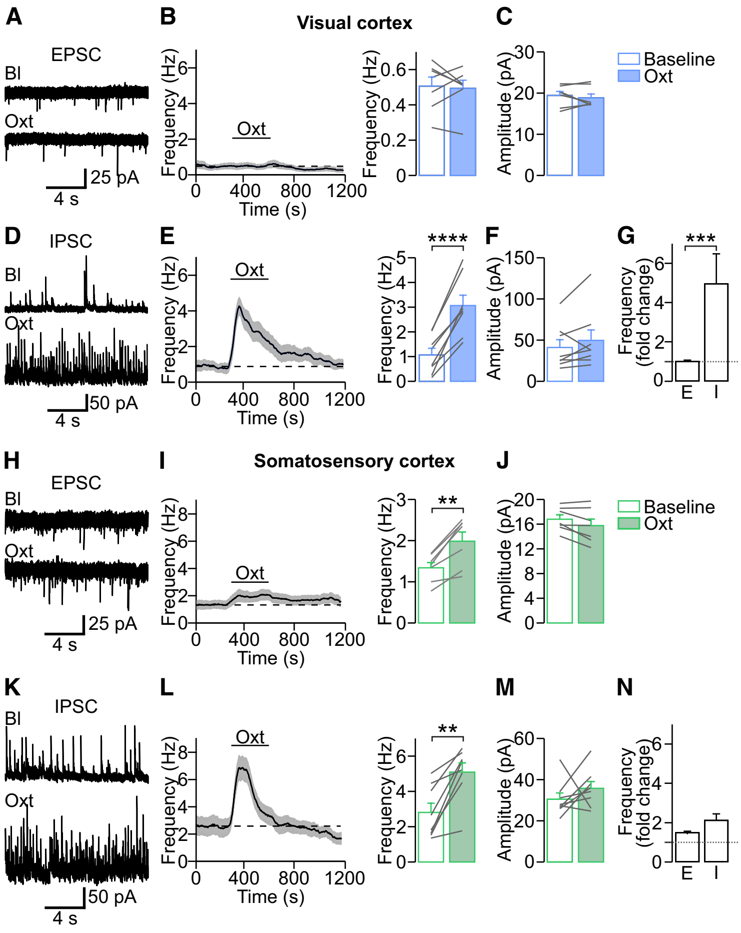

Figure 4. Oxytocin Affects Synaptic Activity Differentially across Sensory Cortices

(A) Voltage-clamp recordings of spontaneous excitatory postsynaptic currents (sEPSCs) during baseline (Bl) and after oxytocin bath application in acute visual cortex slices.

(B) Frequency of sEPSCs before, during, and after oxytocin application. In the visual cortex, oxytocin did not affect EPSC frequency. $p > 0.05$ (n = 7 cells; paired two-tailed t test).

(C) Oxytocin did not affect sEPSC amplitude. $p > 0.05$ (n = 7 cells; paired two-tailed t test).

(D) Spontaneous inhibitory postsynaptic currents (sIPSCs) before and after oxytocin application.

(E) Oxytocin led to a strong increase in sIPSCs. ****$p = 8.2 \times 10^{-5}$ (n = 8 cells; paired two-tailed t test).

(F) Oxytocin did not affect the amplitude of sIPSCs. $p > 0.05$ (n = 8 cells; paired two-tailed t test).

(G) In the visual cortex, oxytocin led to a 5 times increase of sIPSCs, but sEPSCs were unaffected. ***$p = 0.0003$ (n = 7 and n = 8 cells for V1 sEPSCs and sIPSCs, respectively; Mann-Whitney test).

(H) Voltage-clamp recordings of sEPSCs before and after oxytocin bath application in somatosensory cortex slices.

(I) Application of oxytocin increased the frequency of sEPSCs in the somatosensory cortex slightly. **$p = 0.0014$ (n = 7; paired two-tailed t test).

(J) Oxytocin did not affect the amplitude of sEPSCs. $p > 0.05$ (n = 7 cells; paired two-tailed t test).

(K) sIPSCs before and after oxytocin application.

(L) Oxytocin led to a transient increase in sIPSCs. **$p = 0.002$ (n = 8 cells; paired two-tailed t test).

(M) Oxytocin did not affect the amplitude of sIPSCs. $p > 0.05$ (n = 8 cells; paired two-tailed t test).

(N) In the somatosensory cortex, oxytocin led to similar increases in sIPSC and sEPSC frequency. $p = 0.11$ (n = 7 and n = 8 cells for S1 sEPSCs and sIPSCs, respectively; unpaired two-tailed t test).

Data are represented as mean ± SEM. See also Figure S2.

results showed that activation of SST+ interneurons is required for the oxytocin-induced increase in inhibition.

Oxytocin Enhances SST+ Neuron Excitability

Our results suggested that oxytocin triggered inward currents in SST+ interneurons (Figures 5E–5G) and that oxytocin-dependent enhancement of SST+ interneuron firing mediated its effect on network activity (Figures 5H–5J). Therefore, we investigated next whether and how oxytocin-induced inward currents enhanced SST+ neuron firing. In current-clamp mode, we injected a constant current to set the membrane potential of V1 layer 2/3 SST+ interneurons to −60 mV in the presence of the transmitter receptor blockers D-AP5, NBQX, and SR95531. Then, we applied oxytocin while keeping the holding current constant. We observed that oxytocin induced a depolarization of 4.5 ± 0.4 mV (Figure 6A), which exhibited the same transient temporal profile as the sIPSC frequency increase shown in Figure 4E. Current-clamp recordings (Figure 6B) revealed an increase in the firing rate of SST+ interneurons after oxytocin application (Figure 6C). We further studied how oxytocin affected the action potential (AP) properties of SST+ interneurons. Oxytocin (1) increased the AP amplitude and overshoot (Figures 6D and S5A; +2.63 ± 0.78 mV and +2.01 ± 0.93 mV, respectively), (2) broadened the AP width (+0.18 ± 0.03 ms at half-maximum; Figures 6D and 6E), and (3) decreased the time required to generate an AP from the onset of current injection (−1.97 ± 0.32 ms; Figure 6F). In addition, oxytocin modulated AP kinetics as it decreased the maximal speed of voltage change (dV/dt; Figures S5B and S5C). Thus, oxytocin increased the firing capacity and AP properties of V1 layer 2/3 SST+ neurons, most likely by depolarizing their resting membrane potential, as described for PV+ interneurons in the hippocampus.[36] Knowing how oxytocin affects SST+ interneuron activity, we used this information to better understand how oxytocin generated the observed distance-dependent change in correlations. We implemented a recurrent spiking neural network based on a multi-layer model of the thalamocortical system (Figure 6G).[37] To mimic the effect of oxytocin, we increased the resting membrane potential of 25% of the inhibitory neurons, corresponding to the population of SST+ interneurons (Figure 6H). This elevation in membrane potential increased the firing rate of SST+ interneurons for identical current injections (Figure 6I) similarly as observed in our data (Figure 6C). Consequently, in the simulated network, firing of excitatory neurons was strongly suppressed. Inhibitory neurons fired slightly less as well, because their excitatory inputs were largely diminished (Figure 6J). In addition, oxytocin strongly decreased the temporal synchronization of excitatory neurons (Figures 6K and 6L), generating a comparable decrease of correlations over distance as in our experimental data (Figures 2G–2J). This result supported the idea that the increased inhibitory drive produced by

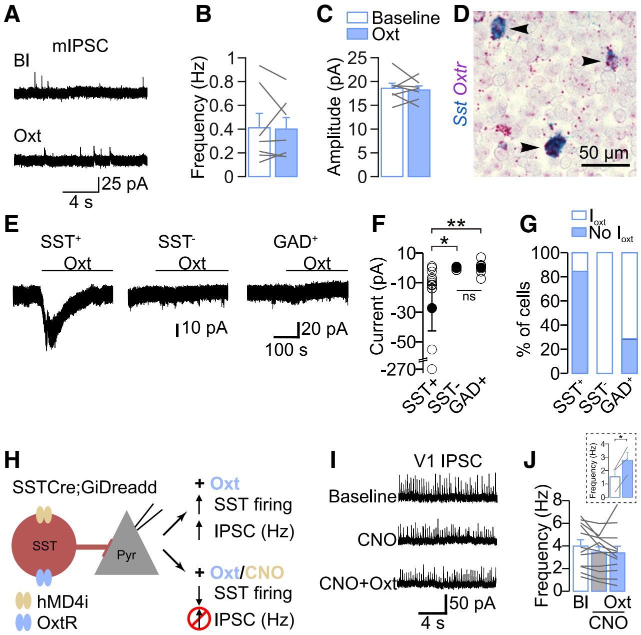

Figure 5. SST^{+} Interneurons Mediate the Oxytocin-Induced Increase in Inhibitory Synaptic Activity in V1

(A) Voltage-clamp recordings of spontaneous miniature synaptic excitatory postsynaptic currents (mIPSCs) in the presence of tetrodotoxin (TTX) (0.5 μM) before and after oxytocin bath application in V1 slices.

(B) The frequency of mIPSCs was not affected by oxytocin. $p > 0.05$ (n = 7 cells; paired two-tailed t test).

(C) The amplitude of mIPSCs was not affected by oxytocin. $p > 0.05$ (n = 7 cells; paired two-tailed t test).

(D) V1 *Oxtr* and *Sst* mRNA transcripts. Note the superposition of the two signals (arrowhead: $Sst^{+}/Oxtr^{+}$ neurons: 93%; $Sst^{-}/Oxtr^{+}$ neurons: 2%).

(E) Examples of voltage-clamp recordings at holding potential of −60 mV from a somatostatin-expressing neuron (SST^{+}) (SST-Cre;Rosa26-TdTomato), an unlabeled neuron (SST^{-}), and a $GAD2^{+}$ neuron ($GAD2^{+}$) (GAD2-Cre;Rosa26-TdTomato) before and after oxytocin application. Oxytocin induced an inward current in the SST^{+} neuron, but not in the unlabeled (most likely excitatory) neuron or in the $GAD2^{+}$ neuron.

(F) Group data of oxytocin-induced inward currents: SST^{+} n = 17 cells; SST^{-} n = 7 cells; and $GAD2^{+}$ n = 7 cells. *p = 0.021; **p = 0.0044. Kruskal-Wallis test, followed by a Dunn test.

(G) Percentage of cells with oxytocin-induced inward currents. Almost all SST^{+} neurons show oxytocin-mediated currents (14/17 cells) but none of the SST^{-} neurons (0/7 cells) and a fraction of $GAD2^{+}$ cells (2/7 cells).

(H) Schematic representation of the experimental paradigm: pyramidal neurons in slices from a transgenic mouse expressing inhibitory DREADDs specifically in SST^{+} interneurons (SST-Cre;Rosa26-Gi-hMD4i) were recorded in voltage-clamp mode. Oxytocin was applied while SST^{+} neuron activity was suppressed by CNO to test whether SST^{+} activation is required for the oxytocin-induced increase in inhibitory synaptic activity.

(I) Example recordings of sIPSCs in baseline condition, in the presence of CNO alone, and after bath application of oxytocin in the presence of CNO.

(J) Oxytocin did not increase sIPSC frequency in the presence of CNO. $p > 0.05$ (n = 12 cells; repeated-measurements one-way ANOVA). Inset: sIPSC frequency is shown. Oxytocin led to an increase in the frequency in the absence of CNO. *p = 0.032 (n = 3 cells; paired two-tailed t test).

Data are represented as mean ± SEM. See also Figures S3 and S4.

oxytocin is sufficient to generate the here-observed effects of oxytocin applications on spontaneous activity patterns, including the distance-dependent changes in interneuronal correlations.

Activation of SST^{+} Interneurons Is Required for Oxytocin-Mediated Modulation of Spontaneous Activity Patterns

Finally, we asked whether the oxytocin-mediated decrease in the frequency of spontaneous network activity was the result of the activation of oxytocin receptors expressed in SST^{+} interneurons *in vivo*. We specifically inactivated SST^{+} interneurons by using a Cre-dependent inhibitory DREADD delivered by virus injection, which results in 80% of the SST^{+} interneurons expressing the hM4Di-DREADD construct.[33] We showed previously that bath application of clozapine *in vitro* reduced the excitability of SST^{+} interneurons,[33] replicating previous findings that hM4Di-DREADD activation reduces the excitability of developing layer 2/3 neurons.[38] We performed *in vivo* wide-field calcium imaging to monitor spontaneous network activity in V1 and then activated the iDREADD receptor by injecting clozapine subcutaneously (Figure 7A). 5 min after clozapine injection, oxytocin was applied topically (Figures 7A and 7B). In this condition, oxytocin failed to decrease the frequency of spontaneous network events compared with oxytocin application alone (Figure 7C, % of change clozapine + oxytocin: −18.1% ± 10.5%; Figure 1D, % of change oxytocin: −49.2% ± 6.2%; p = 0.023; unpaired two-tailed t test). Area, amplitude, and duration were not affected either (Figures 7D–7F). Therefore, SST^{+} neurons are required for the inhibitory effect of exogenous oxytocin on the frequency of spontaneous activity in the developing visual cortex.

We demonstrated here that oxytocin receptor activation can modulate spontaneous activity patterns by specifically activating SST^{+} interneurons; however, the source of oxytocin was exogenous. To evaluate whether endogenous oxytocin indeed modulates spontaneous activity patterns under physiological conditions, we performed wide-field *in vivo* calcium imaging recordings in awake animals, when SST^{+} interneurons are intrinsically active (SST^{+} interneurons are largely silent under anesthesia).[32] When we blocked oxytocin receptors, we observed a small but significant increase in the frequency of calcium events (Figure 7G), whereas the amplitude remained unchanged (Figure 7H), as predicted based on our results after topical application of exogenous oxytocin. This result suggested that the oxytocin receptor is activated by its endogenous ligand and modulates spontaneous activity patterns. Finally, we verified that focal oxytocin application modulates spontaneous activity in the awake condition as well. As in anesthetized animals, we found that the frequency of network events was decreased although their amplitude remained unaffected (mean baseline frequency: 0.047 ± 0.007; mean oxytocin frequency: 0.036 ± 0.005; p = 0.043; paired two-tailed t test; n = 6 animals).

DISCUSSION

In adults, oxytocin is a potent modulator of brain activity and behavior, and it is important for brain development. Here, we demonstrate that, before eye opening, during the 2nd postnatal week, oxytocin modulates specific characteristics of spontaneous activity patterns in the visual cortex: it selectively increases SST^{+} interneuron excitability through oxytocin receptor activation and sparsifies and decorrelates neuronal activity in layer 2/3 of V1 without affecting event area, amplitude, or duration.

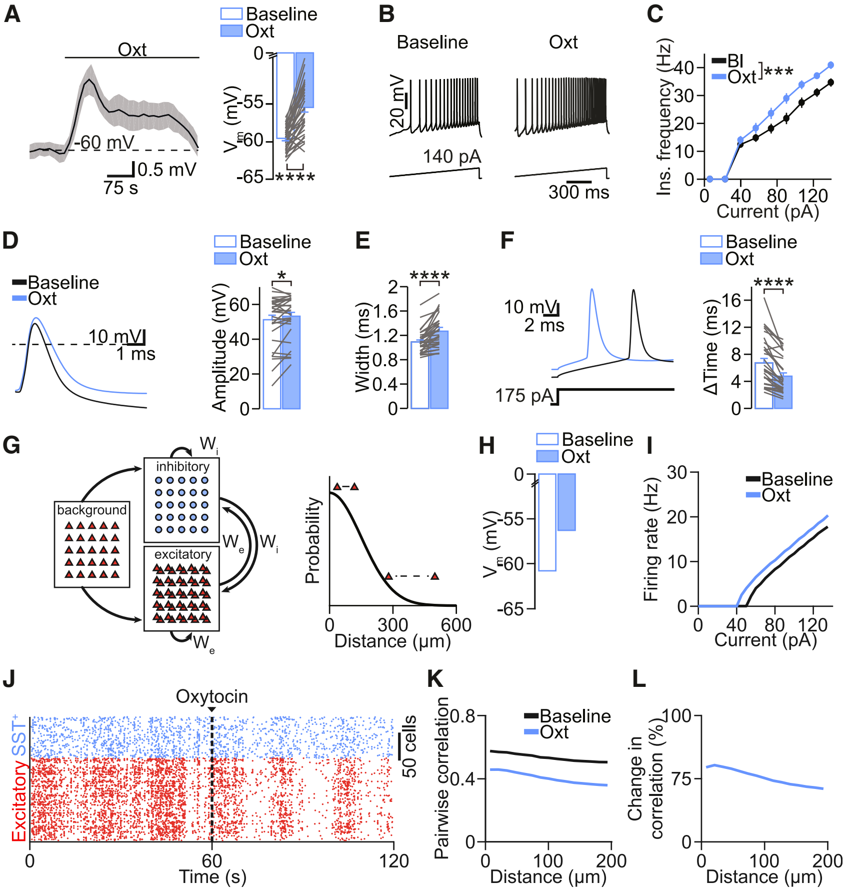

Figure 6. Oxytocin-Induced Increase of the Excitability of SST+ Interneurons Is Sufficient to Explain Distance-Dependent Changes in Correlation

(A) Average of current-clamp recordings of SST^+ neurons before and after oxytocin bath application. Right: group data of SST^+ interneuron membrane potential in baseline and oxytocin conditions are shown. ****p = 4.7 × 10^{-10} (n = 34 cells; paired two-tailed t test).

(B) Train of APs generated with a ramp protocol in current-clamp mode in baseline and oxytocin conditions.

(C) Instantaneous frequency versus current plot. ***p < 0.0001 (n = 34 cells; repeated-measurements two-way ANOVA).

(D) Left: single APs aligned to the peak in baseline and oxytocin conditions. Right: group data of AP amplitude in baseline and oxytocin conditions are shown. *p = 0.038 (n = 34 cells; paired two-tailed t test).

(E) AP width. ****p = 1.8 × 10^{-6} (paired two-tailed t test).

(F) AP. Left: single APs in response to a square current step in baseline and oxytocin conditions. The time required to elicit an action potential was strongly reduced in the presence of oxytocin. ****p = 8.9 × 10^{-7} (paired two-tailed t test). Data are represented as mean ± SEM.

(G) Left: schematic of the model with excitatory background input and recurrently connected excitatory (red) and inhibitory populations (blue). W_e, excitatory weight; W_i, inhibitory weight. Right: connection probability as a function of distance between cells is shown.

(H) Resting membrane potential of SST^+ interneurons during baseline and oxytocin.

(I) Firing rate of inhibitory cells as a function of the input current in baseline and oxytocin.

(J) Spike raster plot of excitatory (red) and SST^+ interneurons (blue) neuron populations before and after application of oxytocin (see STAR Methods).

(K) Correlation as a function of distance for baseline and oxytocin conditions. See STAR Methods for calculation details.

(L) Change in correlation as a function of distance.

See also Figure S5 and Table S1.

The Effect of Oxytocin Receptor Activation Differs between V1 and S1

A large body of evidence suggests that maternal care behaviors induce activation of hypothalamic neurons and release of oxytocin in the pup's brain (e.g., during mother-pup skin-to-skin contact,[39] anogenital stimulation,[40] or stroking stimuli,[41] and most likely after milk suckling activity[42]). Accordingly, somatosensory stimuli specifically activate parvocellular oxytocin neurons in the paraventricular nucleus (PVN).[43] How oxytocin reaches the developing cortex is not entirely clear. It might be released within V1, because hypothalamic oxytocin neurons project to the cortex, including V1, at least in adult mice.[17,44] Alternatively, oxytocin may diffuse into the developing cortex after somatodendritic release from the hypothalamus into the third ventricle.[15,28,42] To fully disentangle how oxytocin reaches the developing cortex and to induce endogenous release, it will be required to adapt technical approaches currently available in adults[18,36,44] to neonatal animals. Nevertheless, our observation that blocking cortical oxytocin receptors increases the frequency of spontaneous network activity in V1 of awake animals indicates that the oxytocin receptor is activated by its endogenous ligand and modulates spontaneous activity patterns during development.

Independently of the source of oxytocin, we find here that this neuropeptide strongly decreases spontaneous network activity in V1, although its effect on spontaneous network activity in S1 is comparably mild. The differences between oxytocin's effect on V1 and S1 network activity are consistent with differences in the expression of its receptor across cell types and their specific responses to oxytocin. Oxytocin receptor mRNA expression co-localizes with the interneuron marker GAD1 in both V1 and S1; however, its expression in excitatory neurons is higher in S1 than V1. Furthermore, oxytocin increases specifically inhibitory synaptic transmission in V1, but in S1, it results in a smaller and more-balanced activation of both spontaneous inhibitory and excitatory currents. Thus, the specific effect of oxytocin on inhibitory signaling is most likely responsible for its effect on network activity patterns in V1. In the adult, various brain regions differ in their responses to oxytocin due to differences in the distribution of the oxytocin receptor in inhibitory neurons as well;[17,18,36,44–47] however, whether oxytocin signaling differs between V1 and S1 in adulthood too is currently unknown.

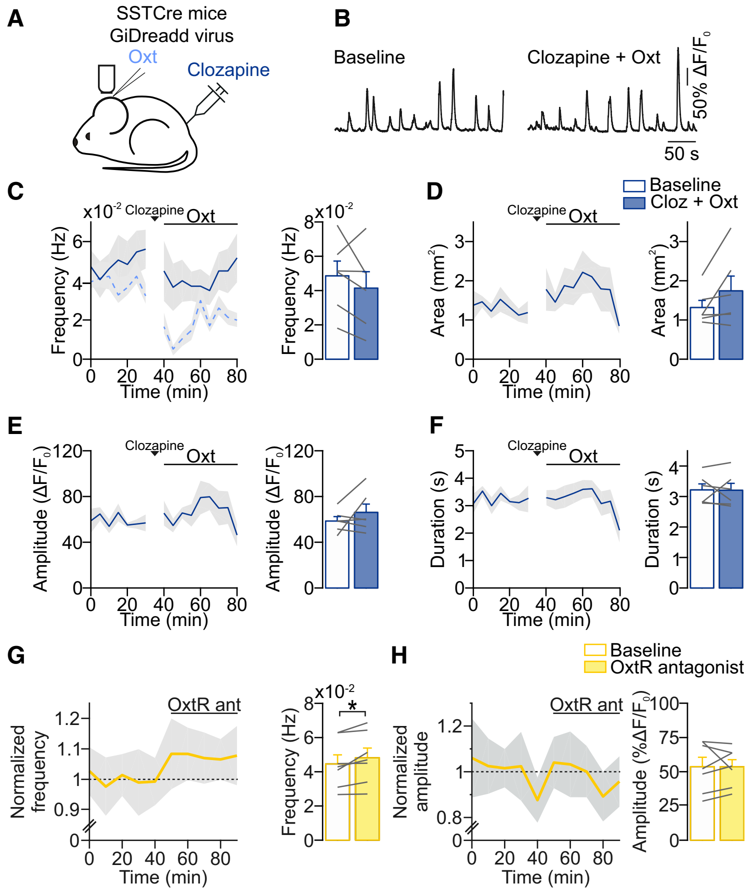

Figure 7. Role for SST+ Interneurons and Endogenous Oxytocin Receptor Activation in Modulating Network Activity *In Vivo*

(A) Schematic representation of the experimental design: wide-field calcium imaging of spontaneous network activity in SST-Cre mice where V1 neurons express GCaMP6s and GiDreadd after viral transduction.

(B) Fluorescent changes before and after clozapine + oxytocin application.

(C) Network event frequency during baseline and after oxytocin application. Time courses represent 5-min averages. Dashed light blue curve represents data shown in Figure 1D, V1, for comparison.

(D) Network event area.

(E) Network event amplitude.

(F) Network event duration. Data are represented as mean ± SEM.

(G) Network event frequency during baseline and after oxytocin receptor antagonist application, in awake animals. *$p = 0.037$ ($n = 7$ animals; paired two-tailed t test). Data are represented as mean ± SEM.

(H) Network event amplitude during baseline and after oxytocin receptor antagonist application, in awake animals.

SST+ Interneuron Activation Accounts for the Oxytocin-Mediated Increase in V1 Inhibitory Transmission

We propose here that the specific activation of SST+ interneurons through oxytocin modulates spontaneous activity patterns in the developing V1 based on five observations: (1) the oxytocin receptor is transcribed in V1 SST+ interneurons but barely detected in other interneuron types, excitatory neurons, or glia cells.[31] (2) Pharmacogenetic suppression of SST+ neurons prevents the oxytocin-mediated decrease in network event frequency *in vivo*. (3) Oxytocin induces an inward current in almost all SST+ interneurons and in a fraction of GAD2+ interneurons. (4) Oxytocin-mediated depolarization of SST+ interneurons increases the frequency of inhibitory synaptic inputs in pyramidal cells 5-fold, and (5) this increase is abolished entirely by SST+ interneuron inactivation.

Our finding that oxytocin increases inhibitory function through SST+ interneuron activation and decreases spontaneous activity during an important step in visual cortex development supports the idea that oxytocin modulation of inhibition facilitates the developmental progression across critical periods.[19,48] During development, changes in the E/I balance are important, because they determine the opening or closure of critical periods, during which sensory pathways become sensitive to experience.[49] Furthermore, the maturation of inhibition may be required for decreasing spontaneous activity,[50] thereby increasing the relative importance of visually evoked activity. This relative increase in experience-driven activity could initiate the critical period 2 weeks after eye opening.[51] Thus, oxytocin may be an important regulator of "phenotypic checkpoints" important for sensitive periods during postnatal development.[52]

Oxytocin Desynchronizes the Network in a Structured Manner

We observed that oxytocin decreases pairwise correlations between neurons. This decorrelation is mediated most likely by oxytocin-induced activation of SST+ interneurons, in agreement with previously published findings: activation of interneurons decreases neuronal correlations in general.[53,54] More specifically, lateral inhibition, a role attributed to SST+ interneurons, decorrelates spike trains[55] or stimulus-evoked patterns,[56] and pharmacogenetic inactivation of SST+ interneurons increases pairwise correlations during spontaneous network activity in the developing visual cortex.[33] Our analyses and simulations suggest that correlations are downregulated in a spatially specific manner: correlation decreases are more pronounced between pairs of neurons that are farther apart. Neuronal correlations and the spatial extent of spontaneous network activity patterns determine their effectiveness in refining synaptic connections in the visual system.[11,25,57,58] Therefore, the regulation of spatial correlations through oxytocin may be necessary to shape spontaneous activity patterns to drive the refinement of synaptic connections and to prepare the emerging network optimally for computing visual inputs after eye opening. It might be tempting to speculate that oxytocin contributes to improved visual acuity after tactile stimulation during development.[59]

During the 2nd postnatal week, V1 activity patterns change from high-correlation, high-cell-participation patterns toward sparser and less-correlated activity. Sparsification of activity patterns has been shown to be largely pre-programmed.[60–62] Because oxytocin signaling ramps up during the 2nd postnatal week and sparsifies activity patterns as discussed above,

oxytocin and potentially other neuromodulators may drive the sparsification and decorrelation seen in spontaneous activity patterns toward the onset of sensation. In this regard, it is interesting to note that activity patterns in the *Fmr1* knockout (KO) mouse, a model for the neurodevelopmental disorder fragile X syndrome, show increased correlations in the developing somatosensory and visual cortices compared to WT littermates.[63,64] In addition, atypical oxytocin signaling has been implicated as a risk factor for neurodevelopmental disorders.[65–67] Thus, oxytocin may be important for healthy brain development to specifically shape activity patterns for synaptic refinement by regulating SST^{+} interneuron function.

STAR★METHODS

Detailed methods are provided in the online version of this paper and include the following:

- KEY RESOURCES TABLE
- RESOURCE AVAILABILITY
  - Lead Contact
  - Materials Availability
  - Data and Code Availability
- EXPERIMENTAL MODEL AND SUBJECT DETAILS
  - Animal
- METHOD DETAILS
  - In utero electroporation and surgery
  - Virus injection
  - Wide-field imaging
  - 2-photon imaging
  - Patch-clamp experiments
  - RNAscope
  - Model
- QUANTIFICATION AND STATISTICAL ANALYSIS
  - 2-photon imaging analysis
  - Wide-field imaging analysis
  - Electrophysiological analysis
  - RNAscope analysis
  - Statistics

SUPPLEMENTAL INFORMATION

Supplemental Information can be found online at https://doi.org/10.1016/j.cub.2020.10.028.

ACKNOWLEDGMENTS

We thank Corette Wierenga, Nicole Ropert, and Marcus Howlett for critically reading the manuscript; Gertjan Houwen for his help with MATLAB coding; Maurice Manning for the donation of the oxytocin receptor antagonist; Monique van Mourik for performing the *in utero* electroporations and virus injection; Fred de Winter for the production of AAV-hSyn-DIO-hM4D(Gi)-mCherry virus; and Marko Popovic for sharing with us the Rapid LED Switching System for one-photon microscopy. We thank the Helmut Kessels lab for sharing equipment, The Allen Brain Institute for permission to reproduce their data in Figure S3, and Marian Verhage for helping with our mice colony. This work was supported by grants of the Netherlands Organization for Scientific Research (NWO, ALW Open Program grants nos. 819.02.017, 822.02.006, and ALWOP.216; ALW Vici, no. 865.12.001), the "Stichting Vrienden van het Herseninstituut" (all C.L.), and the Max Planck Society and European Research Council StG 804824 (J.G.).

AUTHOR CONTRIBUTIONS

Conceptualization, P.P.M. and C.L.; Experiments, P.P.M., A.N.-P., and E.H.; Analysis, P.P.M., A.N.-P., J.K., and J.G.; Writing, P.P.M. and C.L.

DECLARATION OF INTERESTS

The authors declare no competing interests.

Received: February 7, 2020
Revised: August 28, 2020
Accepted: October 9, 2020
Published: November 5, 2020

REFERENCES

1. Katz, L.C., and Shatz, C.J. (1996). Synaptic activity and the construction of cortical circuits. Science *274*, 1133–1138.
2. Sanes, J.R., and Yamagata, M. (2009). Many paths to synaptic specificity. Annu. Rev. Cell Dev. Biol. *25*, 161–195.
3. Kirkby, L.A., Sack, G.S., Firl, A., and Feller, M.B. (2013). A role for correlated spontaneous activity in the assembly of neural circuits. Neuron *80*, 1129–1144.
4. Meister, M., Wong, R.O.L., Baylor, D.A., and Shatz, C.J. (1991). Synchronous bursts of action potentials in ganglion cells of the developing mammalian retina. Science *252*, 939–943.
5. Feller, M.B., Wellis, D.P., Stellwagen, D., Werblin, F.S., and Shatz, C.J. (1996). Requirement for cholinergic synaptic transmission in the propagation of spontaneous retinal waves. Science *272*, 1182–1187.
6. Ackman, J.B., Burbridge, T.J., and Crair, M.C. (2012). Retinal waves coordinate patterned activity throughout the developing visual system. Nature *490*, 219–225.
7. Siegel, F., Heimel, J.A., Peters, J., and Lohmann, C. (2012). Peripheral and central inputs shape network dynamics in the developing visual cortex in vivo. Curr. Biol. *22*, 253–258.
8. Colonnese, M.T., Shen, J., and Murata, Y. (2017). Uncorrelated neural firing in mouse visual cortex during spontaneous retinal waves. Front. Cell. Neurosci. *11*, 289.
9. Weliky, M., and Katz, L.C. (1997). Disruption of orientation tuning in visual cortex by artificially correlated neuronal activity. Nature *386*, 680–685.
10. Cang, J., Rentería, R.C., Kaneko, M., Liu, X., Copenhagen, D.R., and Stryker, M.P. (2005). Development of precise maps in visual cortex requires patterned spontaneous activity in the retina. Neuron *48*, 797–809.
11. Burbridge, T.J., Xu, H.-P., Ackman, J.B., Ge, X., Zhang, Y., Ye, M.-J., Zhou, Z.J., Xu, J., Contractor, A., and Crair, M.C. (2014). Visual circuit development requires patterned activity mediated by retinal acetylcholine receptors. Neuron *84*, 1049–1064.
12. Tyzio, R., Nardou, R., Ferrari, D.C., Tsintsadze, T., Shahrokhi, A., Eftekhari, S., Khalilov, I., Tsintsadze, V., Brouchoud, C., Chazal, G., et al. (2014). Oxytocin-mediated GABA inhibition during delivery attenuates autism pathogenesis in rodent offspring. Science *343*, 675–679.
13. Hanganu, I.L., Staiger, J.F., Ben-Ari, Y., and Khazipov, R. (2007). Cholinergic modulation of spindle bursts in the neonatal rat visual cortex in vivo. J. Neurosci. *27*, 5694–5705.
14. Sarro, E.C., Wilson, D.A., and Sullivan, R.M. (2014). Maternal regulation of infant brain state. Curr. Biol. *24*, 1664–1669.
15. Zheng, J.-J., Li, S.-J., Zhang, X.-D., Miao, W.-Y., Zhang, D., Yao, H., and Yu, X. (2014). Oxytocin mediates early experience-dependent cross-modal plasticity in the sensory cortices. Nat. Neurosci. *17*, 391–399.
16. Hammock, E.A., and Levitt, P. (2013). Oxytocin receptor ligand binding in embryonic tissue and postnatal brain development of the C57BL/6J mouse. Front. Behav. Neurosci. *7*, 195.
17. Mitre, M., Marlin, B.J., Schiavo, J.K., Morina, E., Norden, S.E., Hackett, T.A., Aoki, C.J., Chao, M.V., and Froemke, R.C. (2016). A distributed

network for social cognition enriched for oxytocin receptors. J. Neurosci. *36*, 2517–2535.

18. Marlin, B.J., Mitre, M., D'amour, J.A., Chao, M.V., and Froemke, R.C. (2015). Oxytocin enables maternal behaviour by balancing cortical inhibition. Nature *520*, 499–504.
19. Grinevich, V., and Stoop, R. (2018). Interplay between oxytocin and sensory systems in the orchestration of socio-emotional behaviors. Neuron *99*, 887–904.
20. Burkett, J.P., Andari, E., Johnson, Z.V., Curry, D.C., de Waal, F.B.M., and Young, L.J. (2016). Oxytocin-dependent consolation behavior in rodents. Science *351*, 375–378.
21. Ferretti, V., Maltese, F., Contarini, G., Nigro, M., Bonavia, A., Huang, H., Gigliucci, V., Morelli, G., Scheggia, D., Managò, F., et al. (2019). Oxytocin signaling in the central amygdala modulates emotion discrimination in mice. Curr. Biol. *29*, 1938–1953.e6.
22. Tyzio, R., Cossart, R., Khalilov, I., Minlebaev, M., Hübner, C.A., Represa, A., Ben-Ari, Y., and Khazipov, R. (2006). Maternal oxytocin triggers a transient inhibitory switch in GABA signaling in the fetal brain during delivery. Science *314*, 1788–1792.
23. Winnubst, J., Cheyne, J.E., Niculescu, D., and Lohmann, C. (2015). Spontaneous activity drives local synaptic plasticity in vivo. Neuron *87*, 399–410.
24. Stosiek, C., Garaschuk, O., Holthoff, K., and Konnerth, A. (2003). In vivo two-photon calcium imaging of neuronal networks. Proc. Natl. Acad. Sci. USA *100*, 7319–7324.
25. Leighton, A.H., and Lohmann, C. (2016). The wiring of developing sensory circuits-from patterned spontaneous activity to synaptic plasticity mechanisms. Front. Neural Circuits *10*, 71.
26. Cossell, L., Iacaruso, M.F., Muir, D.R., Houlton, R., Sader, E.N., Ko, H., Hofer, S.B., and Mrsic-Flogel, T.D. (2015). Functional organization of excitatory synaptic strength in primary visual cortex. Nature *518*, 399–403.
27. Ko, H., Cossell, L., Baragli, C., Antolik, J., Clopath, C., Hofer, S.B., and Mrsic-Flogel, T.D. (2013). The emergence of functional microcircuits in visual cortex. Nature *496*, 96–100.
28. Chini, B., Verhage, M., and Grinevich, V. (2017). The action radius of oxytocin release in the mammalian CNS: from single vesicles to behavior. Trends Pharmacol. Sci. *38*, 982–991.
29. Schorscher-Petcu, A., Sotocinal, S., Ciura, S., Dupré, A., Ritchie, J., Sorge, R.E., Crawley, J.N., Hu, S.-B., Nishimori, K., Young, L.J., et al. (2010). Oxytocin-induced analgesia and scratching are mediated by the vasopressin-1A receptor in the mouse. J. Neurosci. *30*, 8274–8284.
30. Manning, M., Misicka, A., Olma, A., Bankowski, K., Stoev, S., Chini, B., Durroux, T., Mouillac, B., Corbani, M., and Guillon, G. (2012). Oxytocin and vasopressin agonists and antagonists as research tools and potential therapeutics. J. Neuroendocrinol. *24*, 609–628.
31. Tasic, B., Menon, V., Nguyen, T.N., Kim, T.K., Jarsky, T., Yao, Z., Levi, B., Gray, L.T., Sorensen, S.A., Dolbeare, T., et al. (2016). Adult mouse cortical cell taxonomy revealed by single cell transcriptomics. Nat. Neurosci. *19*, 335–346.
32. Adesnik, H., Bruns, W., Taniguchi, H., Huang, Z.J., and Scanziani, M. (2012). A neural circuit for spatial summation in visual cortex. Nature *490*, 226–231.
33. Leighton, A.H., Houwen, G.J., Cheyne, J.E., Maldonado, P.P., De Winter, F., and Lohmann, C. (2020). Control of spontaneous activity patterns by inhibitory signaling in the developing visual cortex. bioRxiv. https://doi.org/10.1101/2020.02.21.959262.
34. van Versendaal, D., and Levelt, C.N. (2016). Inhibitory interneurons in visual cortical plasticity. Cell. Mol. Life Sci. *73*, 3677–3691.
35. Gonchar, Y., Wang, Q., and Burkhalter, A. (2008). Multiple distinct subtypes of GABAergic neurons in mouse visual cortex identified by triple immunostaining. Front. Neuroanat. *1*, 3.
36. Tirko, N.N., Eyring, K.W., Carcea, I., Mitre, M., Chao, M.V., Froemke, R.C., and Tsien, R.W. (2018). Oxytocin transforms firing mode of CA2 hippocampal neurons. Neuron *100*, 593–608.e3.
37. Hill, S., and Tononi, G. (2005). Modeling sleep and wakefulness in the thalamocortical system. J. Neurophysiol. *93*, 1671–1698.
38. Naskar, S., Narducci, R., Balzani, E., Cwetsch, A.W., Tucci, V., and Cancedda, L. (2019). The development of synaptic transmission is time-locked to early social behaviors in rats. Nat. Commun. *10*, 1195.
39. Kojima, S., Stewart, R.A., Demas, G.E., and Alberts, J.R. (2012). Maternal contact differentially modulates central and peripheral oxytocin in rat pups during a brief regime of mother-pup interaction that induces a filial huddling preference. J. Neuroendocrinol. *24*, 831–840.
40. Caba, M., Rovirosa, M.J., and Silver, R. (2003). Suckling and genital stroking induces Fos expression in hypothalamic oxytocinergic neurons of rabbit pups. Brain Res. Dev. Brain Res. *143*, 119–128.
41. Okabe, S., Yoshida, M., Takayanagi, Y., and Onaka, T. (2015). Activation of hypothalamic oxytocin neurons following tactile stimuli in rats. Neurosci. Lett. *600*, 22–27.
42. Grinevich, V., Desarménien, M.G., Chini, B., Tauber, M., and Muscatelli, F. (2015). Ontogenesis of oxytocin pathways in the mammalian brain: late maturation and psychosocial disorders. Front. Neuroanat. *8*, 164.
43. Tang, Y., Benusiglio, D., Lefevre, A., Hilfiger, L., Althammer, F., Bludau, A., Hagiwara, D., Baudon, A., Darbon, P., Schimmer, J., et al. (2020). Social touch promotes interfemale communication via activation of parvocellular oxytocin neurons. Nat. Neurosci. *23*, 1125–1137.
44. Knobloch, H.S., Charlet, A., Hoffmann, L.C., Eliava, M., Khrulev, S., Cetin, A.H., Osten, P., Schwarz, M.K., Seeburg, P.H., Stoop, R., and Grinevich, V. (2012). Evoked axonal oxytocin release in the central amygdala attenuates fear response. Neuron *73*, 553–566.
45. Mühlethaler, M., Charpak, S., and Dreifuss, J.J. (1984). Contrasting effects of neurohypophysial peptides on pyramidal and non-pyramidal neurones in the rat hippocampus. Brain Res. *308*, 97–107.
46. Owen, S.F., Tuncdemir, S.N., Bader, P.L., Tirko, N.N., Fishell, G., and Tsien, R.W. (2013). Oxytocin enhances hippocampal spike transmission by modulating fast-spiking interneurons. Nature *500*, 458–462.
47. Nakajima, M., Görlich, A., and Heintz, N. (2014). Oxytocin modulates female sociosexual behavior through a specific class of prefrontal cortical interneurons. Cell *159*, 295–305.
48. Hammock, E.A.D. (2015). Developmental perspectives on oxytocin and vasopressin. Neuropsychopharmacology *40*, 24–42.
49. Hensch, T.K. (2005). Critical period plasticity in local cortical circuits. Nat. Rev. Neurosci. *6*, 877–888.
50. Colonnese, M.T. (2014). Rapid developmental emergence of stable depolarization during wakefulness by inhibitory balancing of cortical network excitability. J. Neurosci. *34*, 5477–5485.
51. Toyoizumi, T., Miyamoto, H., Yazaki-Sugiyama, Y., Atapour, N., Hensch, T.K., and Miller, K.D. (2013). A theory of the transition to critical period plasticity: inhibition selectively suppresses spontaneous activity. Neuron *80*, 51–63.
52. Ben-Ari, Y., and Spitzer, N.C. (2010). Phenotypic checkpoints regulate neuronal development. Trends Neurosci. *33*, 485–492.
53. Cardin, J.A. (2018). Inhibitory interneurons regulate temporal precision and correlations in cortical circuits. Trends Neurosci. *41*, 689–700.
54. Giridhar, S., Doiron, B., and Urban, N.N. (2011). Timescale-dependent shaping of correlation by olfactory bulb lateral inhibition. Proc. Natl. Acad. Sci. USA *108*, 5843–5848.
55. Arevian, A.C., Kapoor, V., and Urban, N.N. (2008). Activity-dependent gating of lateral inhibition in the mouse olfactory bulb. Nat. Neurosci. *11*, 80–87.
56. Friedrich, R.W., and Laurent, G. (2001). Dynamic optimization of odor representations by slow temporal patterning of mitral cell activity. Science *291*, 889–894.
57. Xu, H.-P., Burbridge, T.J., Chen, M.-G., Ge, X., Zhang, Y., Zhou, Z.J., and Crair, M.C. (2015). Spatial pattern of spontaneous retinal waves instructs retinotopic map refinement more than activity frequency. Dev. Neurobiol. *75*, 621–640.

CellPress
OPEN ACCESS

58. Wosniak, M.E., Kirchner, J.H., Chao, L.-Y., Zabouri, N., Lohmann, C., and Gjorgjieva, J. (2020). Adaptive spontaneous activity in the developing visual cortex. bioRxiv. https://doi.org/10.1101/2020.07.30.229559.

59. Guzzetta, A., Baldini, S., Bancale, A., Baroncelli, L., Ciucci, F., Ghirri, P., Putignano, E., Sale, A., Viegi, A., Berardi, N., et al. (2009). Massage accelerates brain development and the maturation of visual function. J. Neurosci. *29*, 6042–6051.

60. Che, A., Babij, R., Iannone, A.F., Fetcho, R.N., Ferrer, M., Liston, C., Fishell, G., and De Marco García, N.V. (2018). Layer I interneurons sharpen sensory maps during neonatal development. Neuron *99*, 98–116.e7.

61. Golshani, P., Gonçalves, J.T., Khoshkhoo, S., Mostany, R., Smirnakis, S., and Portera-Cailliau, C. (2009). Internally mediated developmental desynchronization of neocortical network activity. J. Neurosci. *29*, 10890–10899.

62. Rochefort, N.L., Garaschuk, O., Milos, R.I., Narushima, M., Marandi, N., Pichler, B., Kovalchuk, Y., and Konnerth, A. (2009). Sparsification of neuronal activity in the visual cortex at eye-opening. Proc. Natl. Acad. Sci. USA *106*, 15049–15054.

63. Cheyne, J.E., Zabouri, N., Baddeley, D., and Lohmann, C. (2019). Spontaneous activity patterns are altered in the developing visual cortex of the Fmr1 knockout mouse. Front. Neural Circuits *13*, 57.

64. Gonçalves, J.T., Anstey, J.E., Golshani, P., and Portera-Cailliau, C. (2013). Circuit level defects in the developing neocortex of fragile X mice. Nat. Neurosci. *16*, 903–909.

65. Hammock, E.A.D., and Young, L.J. (2006). Oxytocin, vasopressin and pair bonding: implications for autism. Philos. Trans. R. Soc. Lond. B Biol. Sci. *361*, 2187–2198.

66. Insel, T.R., O'Brien, D.J., and Leckman, J.F. (1999). Oxytocin, vasopressin, and autism: is there a connection? Biol. Psychiatry *45*, 145–157.

67. Muscatelli, F., Desarménien, M.G., Matarazzo, V., and Grinevich, V. (2018). Oxytocin signaling in the early life of mammals: link to neurodevelopmental disorders associated with ASD. Curr. Top. Behav. Neurosci. *35*, 239–268.

68. Evangelidis, G.D., and Psarakis, E.Z. (2008). Parametric image alignment using enhanced correlation coefficient maximization. IEEE Trans. Pattern Anal. Mach. Intell. *30*, 1858–1865.

69. Diesmann, M., and Gewaltig, M.-O. (2002). NEST: An Environment for Neural Systems Simulations (Beiträge zum Heinz-Billing-Preis), pp. 43–70.

70. Pologruto, T.A., Sabatini, B.L., and Svoboda, K. (2003). ScanImage: flexible software for operating laser scanning microscopes. Biomed. Eng. Online *2*, 13.

71. Battefeld, A., Popovic, M.A., van der Werf, D., and Kole, M.H.P. (2019). A versatile and open-source rapid LED switching system for one-photon imaging and photo-activation. Front. Cell. Neurosci. *12*, 530.

72. Chen, T.-W., Wardill, T.J., Sun, Y., Pulver, S.R., Renninger, S.L., Baohan, A., Schreiter, E.R., Kerr, R.A., Orger, M.B., Jayaraman, V., et al. (2013). Ultrasensitive fluorescent proteins for imaging neuronal activity. Nature *499*, 295–300.

STAR★METHODS

KEY RESOURCES TABLE

REAGENT or RESOURCE	SOURCE	IDENTIFIER
'Bacterial and Virus Strains		
pAAV1-hSyn-DIO-hM4D(Gi)-mCherry	[33]	N/A
pAAV1.Syn.GCaMP6s.WPRE.SV40	UPenn viral core	AV-1-PV2824
Chemicals, Peptides, and Recombinant Proteins		
Oregon Green 488 BAPTA-1 AM	Invitrogen	Thermo Fisher Scientific #O6807
Oxytocin	Sigma	O6379
desGly-NH_2,d$(CH_2)_5$[D-Tyr2,Thr4]OVT	Synthesized and given by Dr. Maurice Manning, University of Toledo[30]	N/A
Clozapine N-oxide	Tocris	#0444
Clozapine	Tocris	#4936
SR95531	Tocris	#1262
NBQX	Tocris	#1044
D-AP5	Tocris	#0103
TTX	Tocris	#1078
Critical Commercial Assays		
RNAscope 2.5HD Duplex Assay	Advanced Cell Diagnostics	#322430
Experimental Models: Organisms/Strains		
Mouse: C57B/6J	Janvier	N/A
Sst^{Cre}	The Jackson Laboratory	013044
Gad2^{Cre}	The Jackson Laboratory	010802
Gt(ROSA)26Sor$^{CAG\text{-}tdTomato}$	The Jackson Laboratory	007908
Oligonucleotides		
Oxtr	Advanced Cell Diagnostics	#411101-C2
Slc17a7, VGLUT1	Advanced Cell Diagnostics	#416631
Gad1	Advanced Cell Diagnostics	#400951
Sst	Advanced Cell Diagnostics	#404631
Recombinant DNA		
pCAGGS-DsRed	Gift from Dr. Christiaan Levelt	N/A
pCAGGS-GCaMP6s	Gift from Dr. Christiaan Levelt	N/A
Software and Algorithms		
Enhanced correlation coefficient algorithm	[68]	N/A
NEST	[69]	RRID:SCR_002963
Model simulations	This paper	https://github.com/comp-neural-circuits/OTmodel
LabView	National Instruments	LabView, RRID:SCR_014325

(*Continued on next page*)

Continued

REAGENT or RESOURCE	SOURCE	IDENTIFIER
ScanImage	http://scanimage.vidriotechnologies.com/display/SIH;jsessionid=06CBF1F1626C333384D74630886127FF[70]	ScanImage, RRID:SCR_014307
NIS-Elements	Nikon	(NIS-Elements, RRID:SCR_014329
ImageJ	https://imagej.net/	ImageJ, RRID:SCR_003070
MATLAB	MathWorks	MATLAB, RRID:SCR_001622
Clampfit	Molecular Devices	pClamp, RRID:SCR_011323
AxoGraph	Axograph Scientific	Axograph, RRID:SCR_014284
IGOR Pro	Wave Metrics	IGOR Pro, RRID:SCR_000325
Prism 7	GraphPad	GraphPad Prism, RRID:SCR_002798
Other		
TEAMSTER (Open-source rapid LED switching system for one-photon imaging and photo-activation)	[71]	https://github.com/Kolelab/Image-analysis

RESOURCE AVAILABILITY

Lead Contact

Further information and requests for resources and reagents may be directed to and will be fulfilled by the Lead Contact, Christian Lohmann, c.lohmann@nin.knaw.nl.

Materials Availability

This study did not generate new unique reagents.

Data and Code Availability

The datasets generated during this study have not been deposited in a public repository but are available from the Lead Contact on request. This study used standard, custom-built MATLAB programmed scripts that are available from the Lead Contact upon request. The code used for the simulations is available at https://github.com/comp-neural-circuits/OTmodel.

EXPERIMENTAL MODEL AND SUBJECT DETAILS

Animal

All experimental procedures were approved by the institutional animal care and use committee of the Royal Netherlands Academy of Arts and Sciences and in agreement with the European Community Directive 2010/63/EU and with the Institutional Animal Care and Use Committee at Florida State University in accordance with state and federal guidelines (Guide for the Care and Use of Laboratory Animals of the National Institutes of Health). We used neonatal C57BL/6J, SST-Cre, SST-Cre;Rosa26-TdTomato and GAD2-Cre;Rosa26-TdTomato males and females mice from postnatal day 9 to 14 (P9-14). The SST-Cre;Rosa26-TdTomato and GAD2-Cre;Rosa26-TdTomato lines were generated by crossing the reporter Rosa26-TdTomato line (The Jackson Laboratory, 007908) with either the SST-Cre (The Jackson Laboratory, 013044) or the GAD2-Cre (The Jackson Laboratory, 010802) lines. Neonatal pups were housed with one mother, with exception of pups coming from in utero electroporation. They were housed with two mothers and the nest trimmed to 6 pups to facilitate the caring behavior of the mothers. Animals were kept in a 12h-12h light/dark cycle with food and water *ad libitum*. All the experiments were performed during the light cycle. No effects related to sex were observed. No animal was excluded from the analysis.

METHOD DETAILS

In utero electroporation and surgery

In utero electroporation was performed as described previously[23]. To perform calcium imaging of layer 2/3 pyramidal cells, GCaMP6s was cloned into pCAGGS (Addgene plasmid 40753[72]) and used in combination with DsRed in pCAGGS for visualization (gift from Christiaan Levelt). Pups were in utero electroporated at embryonic day (E) 16.5 after injection of the GCaMP6s (2 μg/μl) and DsRed (1 μg/μl) vectors into the ventricles. Electrode paddles were positioned to target the subventricular zone and 50 V pulses of 50 ms duration were applied.

For *in vivo* experiments, surgery for craniotomy was performed as described previously[7,33]. Pups were kept at 36-37°C and anesthetized with 2% isoflurane and lidocaine was applied into the skin before neck muscle removal. A head bar was fixed above the V1/S1 region. Isoflurane was dropped to 0.7% before the imaging session. This lightly anesthetized state, which is characterized by rapid and shallow breathing and a relatively high heart rate, was maintained throughout the imaging session. During experiments in the absence of anesthesia, pups were kept undisturbed in the dark and covered by a custom-made holder that prevents heat loss and mimic the conditions in the nest, for one hour before the imaging session started. The welfare of the pup was monitored to minimize distress during the whole imaging session.

Virus injection

Virus injections were performed at P0-1. Virus injected were pAAV1-Syn-GCaMP6s (AV-1-PV2824, UPenn viral core) and pAAV1-hSyn-DIO-hM4D(Gi)-mCherry (produced by Fred Winter[33]). SST-Cre neonatal mice were anesthetized by cold-induced hypothermia and kept cold in a stereotactic frame for pups (RWD Life Science). Stereotactic injections targeting V1 were performed with a microinjection pipet (Nanoject II, Drummond; volume 27 nl; mix of 1:1 AAV1-hSyn-DIO-hM4D(Gi)-mCherry and AAV1-Syn-GCaMP6; from Bregma in mm: 0.3 posterior, 1.4 lateral). Immediately after injection, pups were kept warm on a heating pad and placed back to their mother after they awoke from anesthesia.

Wide-field imaging

In utero electroporated or virus injected pups were used for calcium imaging of visual and somatosensory cortex. Calcium events were recorded with a Movable Objective Microscope (MOM, Sutter Instrument). Time-lapse recordings were acquired with a 4x objective (0.8 NA, Olympus) and blue light excitation from a Xenon Arc lamp (Lambda LS, Sutter Instrument Company). A CCD camera (Evolution QEi, QImaging) was controlled by custom-made LabVIEW (National Instruments) based software and images were acquired at a frame rate of 20 Hz.

Clozapine (Tocris) was injected subcutaneously (0.5 mg/kg) and oxytocin was applied five minutes after clozapine injection.

2-photon imaging

Bolus load of the calcium indicator Oregon Green 488 BAPTA-1 AM (OGB-1, Invitrogen) was performed as described[7]. Imaging was performed by using a two-photon microscope (MOM, Sutter, or A1RMP, Nikon) and a mode-locked Ti:Sapphire laser (MaiTai, Spectra Physics or Chamaleon, Coherent, λ = 810 nm). Consecutive xyt-stacks were acquired at a frame rate of 4-7 Hz (pixel size 0.3 - 0.6 μm) through a 40x (0.8 NA, Olympus) or a 16x (0.8 NA, Nikon) water-immersion objective, controlled by ScanImage[70] or NIS-Elements AR4.51.00 software (Nikon).

For *in vivo* experiments, oxytocin (1 μM, Sigma) and the oxytocin receptor antagonist (desGly-NH_2,d$(CH_2)_5$[D-Tyr2,Thr4]OVT, 250 μM, synthesized and kindly donated by Dr. Maurice Manning, University of Toledo) was diluted in cortex buffer solution[7] and applied topically at the craniotomy. The craniotomy was filled with approximately 300 μl of solution and that volume was kept constant until the end of the experiment.

Patch-clamp experiments

Acute 300 μm coronal slices of the visual or somatosensory cortex were dissected between P9-P14. Pups were sacrificed by decapitation and their brains were immersed in ice-cold cutting solution (in mM): 2.5 KCl, 1.25 NaH_2PO_4, 26 $NaHCO_3$, 20 Glucose, 215 Sucrose, 1 $CaCl_2$, 7 $MgCl_2$ (Sigma), pH 7.3-7.4, bubbled with 95%/5% O_2/CO_2. Slices were obtained with a vibratome (Microm HM 650V, Thermo Scientific) and subsequently incubated at 34°C in artificial cerebrospinal fluid (ACSF, in mM): 125 NaCl, 3.5 KCl, 1.25 NaH_2PO_4, 26 $NaHCO_3$, 20 Glucose, 2 $CaCl_2$, 1 MgCl2 (Sigma), pH 7.3-7.4. After 45 minutes, slices were transferred to the electrophysiology setup, kept at room temperature and bubbled with 95%/5% O_2/CO_2. For patch-clamp recordings, slices were transferred to a recording chamber and perfused (3 ml/min) with ACSF solution bubbled with 95%/5% O_2/CO_2 at 34°C.

Layer 2/3 pyramidal cells and interneurons were identified using an IR-DIC video microscope (Olympus BX51WI). $GAD2^+$ and SST^+ interneurons were identified by the TdTomato protein fluorescence from the transgenic mice GAD2-Cre;Rosa26-TdTomato and SST-Cre;Rosa26-TdTomato, respectively. Quick change between bright-field imaging and epifluorescence was achieved using a footswitch device TEAMSTER[71]. Whole-cell voltage or current-clamp recordings were made with a MultiClamp 700B amplifier (Molecular Devices), filtered with a low pass Bessel filter at 10 kHz and digitized at 20-50 kHz (Digidata 1440A, Molecular Devices). Series resistance was assessed during recordings and neurons showing a series resistance > 30 MΩ or a change > 30% were discarded. Digitized data were analyzed offline using Clampfit 10 (Molecular Devices), Igor (WaveMetrics) and AxoGraph (Axograph Scientific).

Spontaneous and miniature IPSCs were recorded at a holding potential of 10 mV with glass pipettes (3-6 MΩ) containing (in mM): 115 $CsCH_3SO_3$, 10 HEPES, 20 CsCl, 2.5 $MgCl_2$, 4 ATP disodium hydrate, 0.4 GTP sodium hydrate, 10 phosphocreatine disodium hydrate, 0.6 EGTA (Sigma), pH 7.3. For sIPSC recordings during selective silencing of SST^+ interneurons, clozapine N-oxide (CNO) (10 μM, Tocris) was administered 1 minute prior to oxytocin (1 μM, Sigma). mIPSCs were recorded in the presence of tetrodotoxin (0.5 μM, Tocris). The oxytocin receptor antagonist desGly-NH_2,d(CH_2)$_5$[D-Tyr2,Thr4]OVT (50 μM, synthesized and kindly donated by Dr. Maurice Manning, University of Toledo) was applied for 5-10 minutes before oxytocin wash-in. sEPSCs were recorded at a holding potential of −60 mV (with junction potential correction) with an intracellular solution containing (in mM): 122 potassium gluconate, 10 HEPES, 13 KCl, 10 phosphocreatine disodium hydrate, 4 ATP magnesium salt, 0.3 GTP sodium hydrate (Sigma), pH 7.3. Oxytocin-induced currents in $GAD2^+$ and SST^+ interneurons were recorded at a holding potential of −60 mV (with junction potential correction) in the presence of 10 μM SR95531, 10 μM NBQX, 50 μM D-AP5 (Tocris). Current-clamp recordings of SST^+ interneurons were performed using the same KGluconate-based intracellular solution and in the presence of the synaptic blockers mentioned above. After breaking the seal, variable current injection was applied to keep the cells at −60 mV. The injected current was kept constant from the time of oxytocin wash-in.

RNAscope

Fresh frozen brain tissue from C57BL/6J mice was sectioned in the sagittal plane at 20 μm in 6 series on SuperFrost Plus microscope slides and stored at −80°C until further processing. RNA transcripts were detected with RNAscope 2.5HD Duplex Assay (Cat. No. 322430, Advanced Cell Diagnostics (ACD), Hayward, CA). Synthetic oligonucleotide probes complementary to the nucleotide sequence 1198 – 2221 of *Oxtr* (NM_001081147.1; ACD Cat. No. 411101-C2), 464 - 1415 of *Slc17a7* (VGLUT1; NM_182993.2; ACD Cat. No. 416631), 62 – 3113 of *Gad1* (NM_008077.4; ACD Cat. No. 400951) and 18 – 407 of *Sst* (NM_009215.1; ACD Cat. No. 404631) were used. Slides were fixed for 2 hours in ice cold 4% paraformaldehyde (pH 9.5) followed by increasing concentrations of ethanol and dehydration in 100% ethanol overnight at −20°C. Slides were air-dried for 10 minutes and boiled for 5 minutes in a target retrieval solution (ref. 322001, ACD), followed by 2 room temperature water rinses and a rinse in 100% ethanol. Slides were air-dried, after which targeted sections were incubated with Protease Plus solution (ref. 322331, ACD) for 15 minutes at 40°C, followed by room temperature water rinses. These prepared slides were then probed for 2 hours with individual probe mixtures (*Oxtr* in the red channel 2 and the other probes in the blue-green channel 1) at 40°C. Unbound probes were rinsed off in wash buffer and slides were stored overnight in 5X SSC at room temperature. Signal amplification and detection were performed using the detailed instructions provided in the RNAscope 2.5HD Duplex Assay. Sections were counterstained with Gill's hematoxylin (American Mastertech Scientific, Inc. Lodi, CA) and coverslipped with Vectamount (Vector Laboratories, Inc. Burlingame, CA). Images were captured with brightfield microscopy (Keyence BZ-X710, Keyence Corp., Osaka, Japan).

Model

We simulated a large-scale computational model of the visual cortex[37] in the NEST framework[69]. We focused only on modeling the cortical network of excitatory and inhibitory neurons with 25% of the inhibitory population corresponding to SST^+ neurons in L2/3, receiving excitatory input from the thalamus. To achieve network activity similar to that described for the developing cortex[7,62], we adjusted the intrinsic membrane properties and synaptic weights in the model based on measurements from the present study (Table S1) and applied an external current of 50 pA (to excitatory neurons) or 40 pA (to SST^+ interneurons) and a sinusoidal background input to the excitatory population to generate spontaneous activity. The oxytocin effect was modeled by increasing the resting membrane potential of SST^+ interneurons from −60.8 mV to −56.3 mV as the change observed in our experiments (Figure 6A). After simulating the system for 60 s in baseline condition, we modeled application of oxytocin and simulated again for 60 s. Correlations are computed between the voltage traces of excitatory units.

QUANTIFICATION AND STATISTICAL ANALYSIS

2-photon imaging analysis

Images were analyzed with ImageJ (NIH) and custom-written MATLAB scripts (MathWorks) as described previously[63]. First, to remove movement artifacts and align all recordings we performed an image alignment step based on the enhanced correlation coefficient algorithm[68]. $\Delta F/F_0$ stacks were generated by subtracting and dividing each frame by the mean fluorescence (F_0). Regions of interest (ROIs) were placed on cells that showed clear activity and were visible in all recordings. Glial cells in the field of view showed elevated basal intensity and were not active. All included ROIs were neuronal. $\Delta F/F_0$ traces were obtained by calculating the mean intensity within the ROI for each frame. Increases in fluorescence intensity, which reflect increases in the intracellular calcium concentration due to action potential firing, were then detected automatically for all ROIs and subsequently verified manually. The detection threshold was adjusted for each experiment (at least 2x the standard deviation of the signal in the absence of events) but remained the same within an experiment.

Pearson correlation coefficients were computed from the $\Delta F/F_0$ stacks. The total number of pairs of cells (computed as $N(N-1)/2$) was balanced across conditions (oxytocin – 8806 pairs; cortex buffer – 8023 pairs). In Figure 2E each x-coordinate corresponds to a window of length 7 minutes around the time point. The y-coordinate of the line is computed as the average across all animals of the condition over the correlation coefficients computed from the window. The shaded area is the standard error of the mean (computed as $\widehat{\sigma}/\sqrt{N}$, where $\widehat{\sigma}$ is the estimated standard deviation over animals and N is the number of animals). In Figure 2F, we averaged the

correlation coefficients computed from the windows over all animals and time points before or after oxytocin application. The filled contour plot was made by plotting pairwise correlations after oxytocin (average of 40 minutes period after oxytocin) against baseline correlations (average of 40 minutes period before oxytocin).

For the matrix correlation analysis, we computed the squared distance between the baseline matrix, c_{ij}^{BL}, computed for the entire baseline period of 45 minutes, and the correlation matrix computed from a shifting 7-minute slice centered at time point t, c_{ij}^{t}, and then summing over all the element-wise differences of all matrix entries, $MSD = \sum_{ij} (c_{ij}^{t} - c_{ij}^{BL})^2$. To remove any potential drift that occurs already during the baseline and that might distort the MSD analysis, we performed a linear regression on the baseline MSD and subtracted the linear contributions from all time points. To further pool across multiple animals, we normalized the squared distances by the mean and standard deviation of the baseline period.

Wide-field imaging analysis

Network events were detected automatically. First, $\Delta F/F_0$ stacks were generated using a moving average across 500 frames as F_0. The detection threshold was 12% $\Delta F/F_0$, which was at least 3x larger than the standard deviation of the noise during inactivity for all recordings. Pixels below threshold were reduced to zero. Network events were defined as groups of at least 300 non-zero pixels that were connected in time and/or space. Since the frame rate was 20 Hz and the pixel size 7.68 μm (area covered by 1 pixel: 59 μm^2), this criterion included events of e.g., > 1 s duration that covered on average > 885 μm^2 of cortical surface. Since this criterion was chosen empirically, we investigated how robust our findings were with respect to this criterion. We performed analyses where we varied the total number of connected pixels that defined a network event on a subset of the data. We found that the number of detected events and the changes observed in frequency after oxytocin application were very robust across different pixel numbers between 200 and 800 pixels (not shown). Since wide-field microscopy is inherently prone to scattered light, we found that the area that network events covered across the cortex was overestimated when we used the 12% cutoff. Therefore, we restricted the area to those pixels that reached 67% of the maximal $\Delta F/F_0$ value for each event (Figure S6).

Recordings from un-anaesthetized animals showed increased movement artifacts. Therefore, we restricted our analysis to frequency and amplitude of network events in traces generated from the entire area of V1. Traces were long-pass filtered to remove high-frequency movement artifacts. Network events were automatically detected using MATLAB's "Find peaks" function where the parameters prominence and peak were optimized for detecting events during the baseline period and kept constant throughout the experiment.

Electrophysiological analysis

m/sIPSCs and sEPSCs were detected using an Igor-based tool SpAcAn (Igor Pro 7, WaveMetrics). Frequency timelines of postsynaptic currents were built by calculating the frequency of 50-or 90 s bins. The 20%–80% rise time was calculated for each IPSC event and the rise rate was determined as amplitude/rise time (pA/ms). Excitability of SST^+ interneurons was assessed with a one-step ramp protocol, from −100 pA to 140 pA at a rate of 96 pA/second. Single action potentials (APs) were elicited by injecting moderate pulses of current (< 1 nA, < 15 ms). Changes in membrane potential upon oxytocin treatment were calculated as the voltage difference between the trace exhibiting the peak effect and the last trace of baseline condition. A minority of cells that did not exhibit a depolarization (6%) were discarded for subsequent analyses. A voltage timeline was built by calculating the baseline membrane potential of 45 s bins. For analysis of SST^+ interneuron excitability, the duration of the ramp was divided into 175-milisecond bins and the mean inter-spike interval (ISI) was calculated for each period bin. Then, the instantaneous frequency (ISI^{-1}) was plotted against the mean current injected within the same bin. AP features were determined as follows: (1) overshoot was quantified as the amplitude of the AP above 0 mV. (2) Amplitude was calculated as the voltage difference between the peak of the AP and its threshold. (3) Width was determined at half the amplitude of the AP. (4) ΔTime was described as the time difference between the onset of pulse injection and the time point when the membrane potential reached the action potential threshold. (5) $dVdt^{-1}$ was defined as the first derivative of the voltage trace with respect to time.

RNAscope analysis

Quantification was done on the 40x images. The total number of cells was determined by the nucleus visualization given by Gill's hematoxalin staining. *Oxtr*$^+$/VGLUT1$^+$/*Gad1*$^+$ neurons were manually identified and counted with ImageJ.

Statistics

All data are shown as mean ± SEM. The number of animals and the test used for each analysis is specified in the Results section. To determine statistical differences we used Prism 7 (GraphPad). Sets of data ≥ 6 were tested for normality with a Shapiro-Wilk test, then a paired or unpaired t test was applied for two-group comparisons. Comparisons between more than two groups were performed with one or two-way ANOVAs. For not normally distributed data or data < 6, the non-parametric Wilcoxon and Mann-Whitney tests were applied for paired or unpaired experiments, respectively, for two-group comparisons. Datasets with more than two groups were analyzed using the Kruskal-Wallis and Friedman test.

Current Biology, Volume *31*

Supplemental Information

Oxytocin Shapes Spontaneous Activity

Patterns in the Developing Visual Cortex

by Activating Somatostatin Interneurons

Paloma P. Maldonado, Alvaro Nuno-Perez, Jan H. Kirchner, Elizabeth Hammock, Julijana Gjorgjieva, and Christian Lohmann

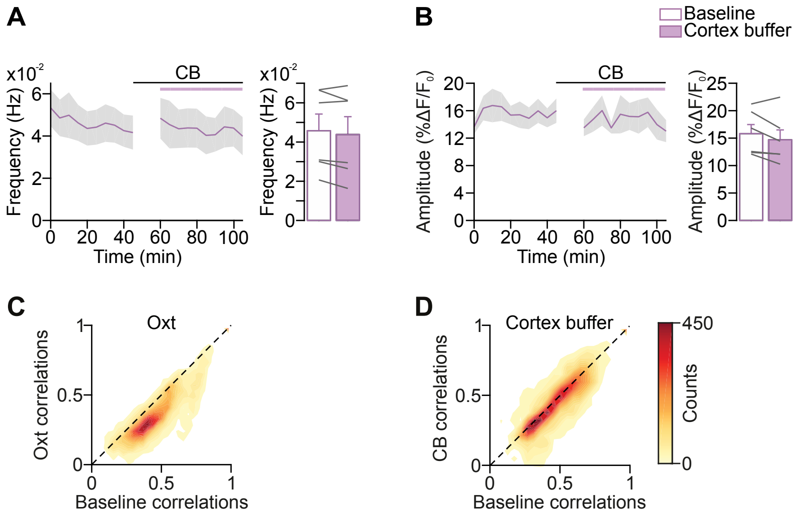

Figure S1. Effect of oxytocin and cortex buffer in two-photon imaging, related to Figure 2.

(A) Network event frequency during baseline and after cortex buffer application. Imaging resumed approximately 10 minutes after cortex buffer application. The frequency of network events was unchanged after cortex buffer application. The horizontal bar indicates significant deviations from baseline as in Figure 1 (dark shades, paired two-tailed t-test, $p < 0.05$, without multi-measurement correction). $P = 0.17$ ($n = 6$ animals, paired two-tailed t-test). CB: cortex buffer.

(B) Network event amplitude during baseline and after cortex buffer application. $P = 0.14$ (paired two-tailed t-test). Data are represented as mean ± SEM.

(C) Filled contour plot of the joint distribution of Pearson correlation coefficients. Pairwise correlations after oxytocin application plotted against baseline correlations.

(D) Pairwise correlations after cortex buffer plotted against baseline correlations.

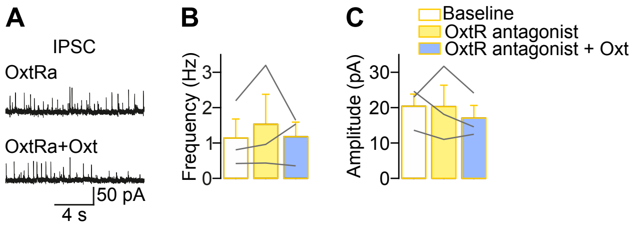

Figure S2. The oxytocin receptor mediates the increase in sIPSC frequency after oxytocin application, related to Figure 4.

(A) V1 sIPSCs in the presence of the oxytocin receptor antagonist (desGly-NH_2,d$(CH2)_5$[D-Tyr^2,Thr^4]OVT, donation from Dr. Maurice Manning, 50 µM) before (top) and after applying oxytocin (bottom).

(B) When oxytocin receptors were blocked, oxytocin failed to increase the frequency of sIPSCs. P = 0.53 (n = 3 cells, Friedman test).

(C) Amplitude of sIPSCs in the presence of the oxytocin receptor antagonist before and after oxytocin application. Data are represented as mean ± SEM.

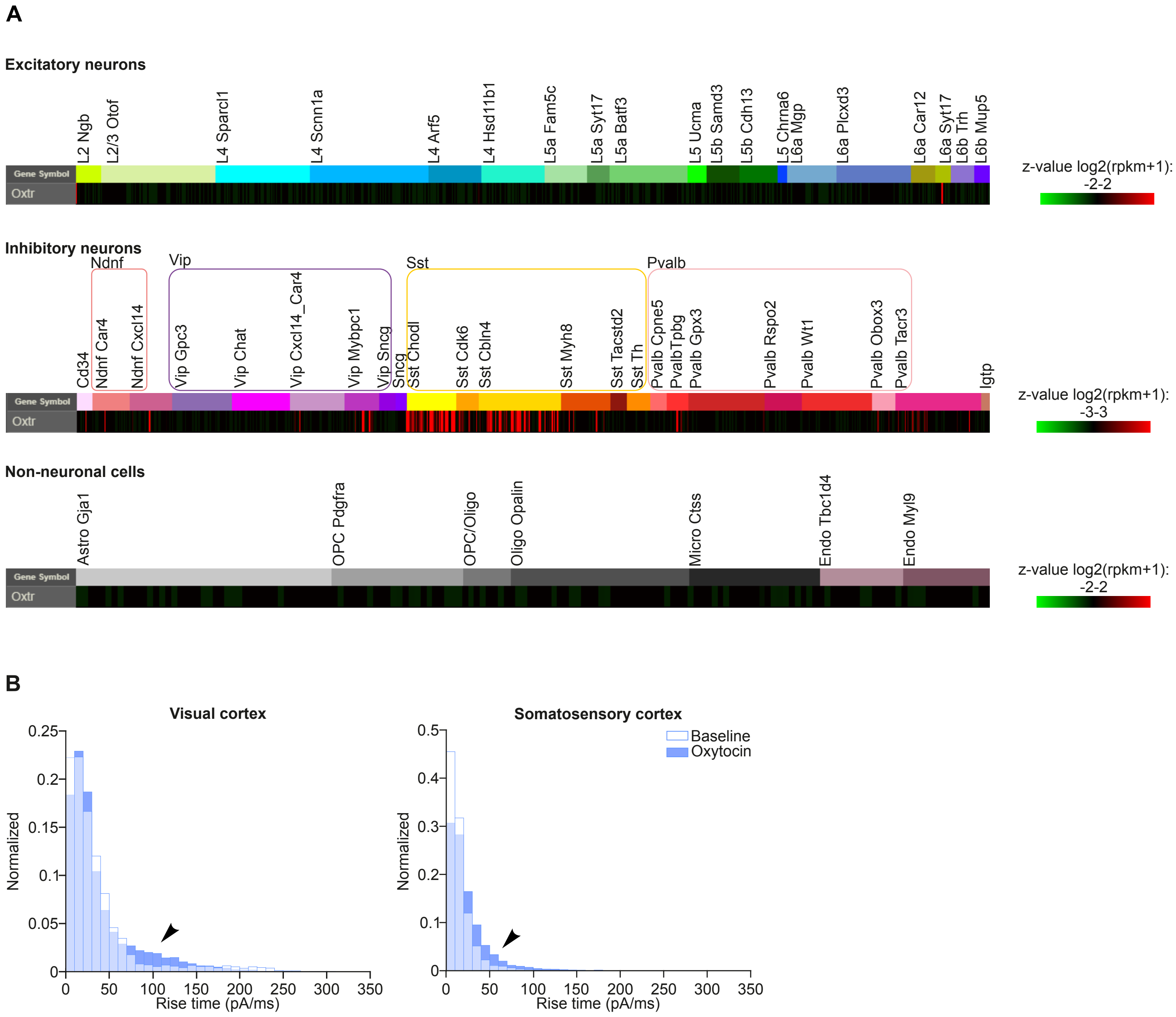

Figure S3. Evidence for oxytocin receptor activation in somatostatin^{+} interneurons , related to Figure 5.

(A) Single cell RNA-sequencing of adult visual cortex for the oxytocin receptor gene (*Oxtr*). *Oxtr* is only expressed in interneurons, in particular in those of the somatostatin-expressing type. Adapted with permission from the Allen Brain Institute [S1], Allen Brain Atlas data portal: http://casestudies.brain-map.org/celltax.

(B) Left, rise time rate histogram of V1 sIPSCs. Oxytocin shifted the histogram to the right. N = 8 cells. Kolmogorov-Smirnov test, $p < 0.0001$. Right, rise time rate histogram of S1 sIPSCs. As for V1, oxytocin shifted the histogram to the right as well. N = 8 cells. Kolmogorov-Smirnov test, $p < 0.0001$.

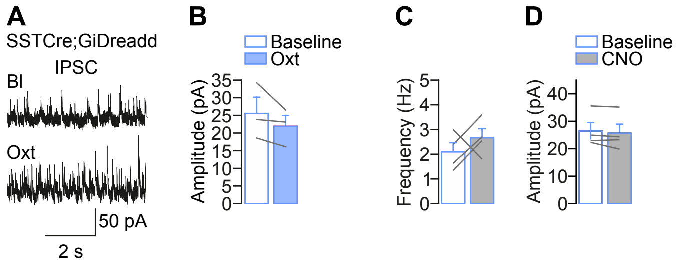

Figure S4. Oxytocin and CNO effect in SSTCre;GiDreadd and WT mice, respectively, related to Figure 5.

(A) sIPSCs before and after oxytocin application from a V1 layer 2/3 pyramidal cell of an SSTCre;GiDreadd mouse in the absence of CNO.

(B) sIPSC amplitude. N = 3 cells.

(C) sIPSC frequency before and after CNO application of WT V1 layer 2/3 pyramidal cells. P = 0.5 (n = 4 cells, Wilcoxon test).

(D) Amplitudes of sIPSCs. Data are represented as mean ± SEM.

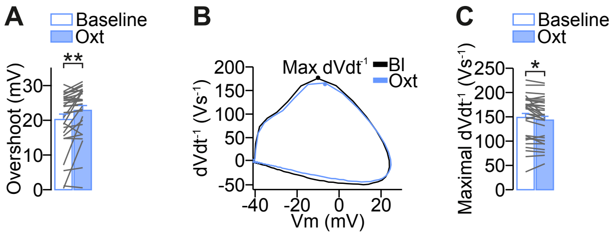

Figure S5. Oxytocin affects single action potential properties of somatostatin⁺ interneurons, related to Figure 6.

(A) AP overshoot in baseline and oxytocin conditions. **P = 0.0032 (n = 34 cells, paired two-tailed t-test).

(B) Example action potential phase plot in baseline and oxytocin conditions.

(C) Maximal time derivative of membrane voltage in baseline and oxytocin conditions. *P = 0.017 (paired two-tailed t-test). Data are represented as mean ± SEM.

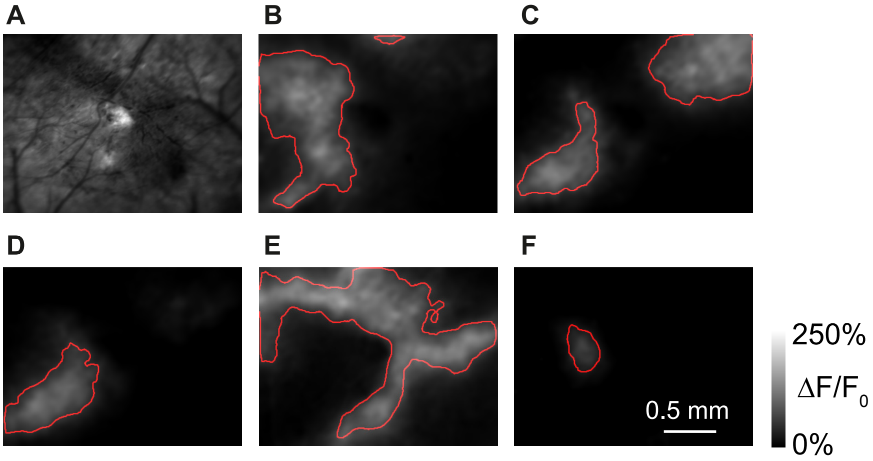

Figure S6. Automated event detection for wide-field imaging, related to Figure 1 and STAR Methods.

(A) Cortical surface showing GCaMP6s labeling.

(B-F) Spontaneous network events represented as %$\Delta F/F_0$ values in single frames and outlines generated by the automated analysis.

**Physiological parameter**	**Value**
Resting membrane potential V_m	-68.0 mV (excitatory)<br>-60.8 mV (SST^+ interneurons)<br>-56.3 mV (SST^+ interneurons after oxytocin)
Membrane time constant τ_m	84.0 ms (excitatory)<br>106.6 (SST^+ interneurons)
Spike threshold V_{th}	-36 mV (excitatory)<br>-33 mV (SST^+ interneurons)
Capacitance C_m	200 pF
Sinusoidal background input	0.05 Hz
Synaptic weight W_e, W_i	8.4 mV (AMPA)<br>-21.3 mV (GABA)

Table S1. Parameters for simulation of recurrent neural network, related to Figure 6.

Supplemental References

S1. Tasic, B., Menon, V., Nguyen, T.N., Kim, T.K., Jarsky, T., Yao, Z., Levi, B., Gray, L.T., Sorensen, S.A., Dolbeare, T., *et al.* (2016). Adult mouse cortical cell taxonomy revealed by single cell transcriptomics. Nat. Neurosci. *19*, 335–346.
